# Distinct DNA methylation signatures associated with blood lipids as exposures or outcomes among survivors of childhood cancer: a report from the St. Jude lifetime cohort

**DOI:** 10.1186/s13148-023-01447-3

**Published:** 2023-02-28

**Authors:** Qian Dong, Cheng Chen, Nan Song, Na Qin, Noel-Marie Plonski, Emily R. Finch, Kyla Shelton, John Easton, Heather Mulder, Emily Plyer, Geoffrey Neale, Emily Walker, Qian Li, I-Chan Huang, Jinghui Zhang, Hui Wang, Melissa M. Hudson, Leslie L. Robison, Kirsten K. Ness, Zhaoming Wang

**Affiliations:** 1 Department of Epidemiology and Cancer Control, St. Jude Children’s Research Hospital, 262 Danny Thomas Place, MS 735, Memphis, TN 38105 USA; 2 School of Public Health, Shanghai Jiaotong University, Shanghai, China; 3 College of Pharmacy, Chungbuk National University, Cheongju, Korea; 4 Department of Epidemiology, Center for Global Health, School of Public Health, Nanjing Medical University, Nanjing, Jiangsu China; 5 Department of Computational Biology, St. Jude Children’s Research Hospital, Memphis, TN USA; 6 Hartwell Center, St. Jude Children’s Research Hospital, Memphis, TN USA; 7 Department of Biostatistics, St. Jude Children’s Research Hospital, Memphis, TN USA; 8 Department of Oncology, St. Jude Children’s Research Hospital, Memphis, TN USA

**Keywords:** DNA methylation, Lipid levels, Childhood cancer survivors, EWAS, African ancestry, European ancestry

## Abstract

**Background:**

DNA methylation (DNAm) plays an important role in lipid metabolism, however, no epigenome-wide association study (EWAS) of lipid levels has been conducted among childhood cancer survivors. Here, we performed EWAS analysis with longitudinally collected blood lipid data from survivors in the St. Jude lifetime cohort study.

**Methods:**

Among 2052 childhood cancer survivors of European ancestry (EA) and 370 survivors of African ancestry (AA), four types of blood lipids, including high-density lipoprotein (HDL), low-density lipoprotein (LDL), total cholesterol (TC), and triglycerides (TG), were measured during follow-up beyond 5-years from childhood cancer diagnosis. For the exposure EWAS (i.e., lipids measured before blood draw for DNAm), the DNAm level was an outcome variable and each of the blood lipid level was an exposure variable; vice versa for the outcome EWAS (i.e., lipids measured after blood draw for DNAm).

**Results:**

Among EA survivors, we identified 43 lipid-associated CpGs in the HDL (*n* = 7), TC (*n* = 3), and TG (*n* = 33) exposure EWAS, and 106 lipid-associated CpGs in the HDL (*n* = 5), LDL (*n* = 3), TC (*n* = 4), and TG (*n* = 94) outcome EWAS. Among AA survivors, we identified 15 lipid-associated CpGs in TG exposure (*n* = 6), HDL (*n* = 1), LDL (*n* = 1), TG (*n* = 5) and TC (*n* = 2) outcome EWAS with epigenome-wide significance (*P* < 9 × 10^−8^). There were no overlapping lipids-associated CpGs between exposure and outcome EWAS among EA and AA survivors, suggesting that the DNAm changes of different CpGs could be the cause or consequence of blood lipid levels. In the meta-EWAS, 12 additional CpGs reached epigenome-wide significance. Notably, 32 out of 74 lipid-associated CpGs showed substantial heterogeneity (*P*_*het*_ < 0.1 or *I*^2^ > 70%) between EA and AA survivors, highlighting differences in DNAm markers of blood lipids between populations with diverse genetic ancestry. Ten lipid-associated CpGs were cis-expression quantitative trait methylation with their DNAm levels associated with the expression of corresponding genes, out of which seven were negatively associated.

**Conclusions:**

We identified distinct signatures of DNAm for blood lipids as exposures or outcomes and between EA and AA survivors, revealing additional genes involved in lipid metabolism and potential novel targets for controlling blood lipids in childhood cancer survivors.

**Supplementary Information:**

The online version contains supplementary material available at 10.1186/s13148-023-01447-3.

## Background

Mounting evidence suggests that epigenetics, specifically DNA methylation (DNAm), plays an important role in lipid metabolism, and epigenome-wide association studies (EWAS) of blood lipid levels have identified robust 5′-cytosine-phosphate-guanine-3′ (CpG) sites and plausible underlying genes associated with lipid metabolism and related diseases [1]. However, an EWAS analysis of lipid levels has not been conducted among survivors of childhood cancer who experience early onset and a substantially higher burden of chronic health conditions (CHCs), compared to community controls without a history of childhood cancer [2, 3]. These health disparities are mostly attributable to genotoxic cancer treatment exposures at a young age with the most notable link being between cardiovascular diseases and exposures to anthracyclines and/or chest-directed radiation therapy (RT) [4]. Recognizing the high burden of CHCs among childhood cancer survivors [2, 3, 5], we have comprehensively analyzed DNAm variations among long-term survivors and conducted systematic investigations of potential casual pathways for treatment-associated CHCs [6]. Our previous findings provide compelling evidence of mediation effect of DNAm between abdominal-RT and dyslipidemia (triglycerides > 150 mg/dL or total cholesterol > 200 mg/dL) [6]. Dyslipidemia is highly prevalent within the broad spectrum of morbidities of childhood and adolescent cancer survivors [7], and a major risk factor for cardiac events, which are the leading cause of noncancer-related premature mortality and account for approximately 26% of deaths among survivors within 45 years of diagnosis [8].

In the general population, African American adults have higher prevalence of high low-density lipoprotein (LDL) and low high-density lipoprotein (HDL) levels but lower prevalence of high triglycerides (TG) than European American adults in both men and women [9, 10]. A study considering racial/ethnic differences among childhood cancer survivors in the St. Jude lifetime cohort study (SJLIFE) reported that childhood cancer survivors of African ancestry (AA) had higher risk of cardiovascular diseases overall including specific conditions such as stroke, heart attack, and heart failure than survivors of European ancestry (EA), potentially explained by the higher prevalence of obesity, diabetes, hypertension, and dyslipidemia among AA survivors [11]. Studies have reported notable population-specific DNAm differences in multiple physical functions (e.g., immunity and kidney development) [12, 13], suggesting that EWAS across populations is critical to the interpretation of health disparities [14]. However, there is a lack of diversity in currently available EWAS data, with most studies conducted in individuals of EA.

To further our understanding of the underlying biological mechanisms of different blood lipid levels among childhood cancer survivors and the differences between EA and AA populations as determined by their genetic ancestry, we employed a comprehensive and agnostic EWAS approach across these two populations. Taking advantage of longitudinal clinical assessments of SJLIFE survivors, we analyzed association of DNAm with blood lipids as exposures (i.e., blood lipids were measured before DNAm) and outcomes (i.e., blood lipids were measured after DNAm). Findings were compared between these two scenarios as well as between the two ancestral groups (i.e., EA and AA). The potential function of significant CpG sites were further demonstrated by their correlations with gene expression levels measured by RNA sequencing. We compared our findings among childhood cancer survivors with the known blood lipid-associated CpGs previously reported in non-cancer general populations. Clinically, the set of lipid-associated CpG sites (i.e., signatures) would facilitate the identification of survivors who have already experienced abnormal lipid levels or at higher risk of abnormal lipid levels in the future.

## Results

### Characteristics of the study population and EWAS analysis design

Adult survivors of childhood cancer from the SJLIFE study [15, 16] were included in this analysis (Table 1). Among 2052 EA survivors (median age at blood draw for DNAm = 32.3 years, interquartile range [IQR] = 26.5–40.1 years; 47.2% female), body mass index (BMI) was 9.9–67.7 kg/m^2^ (Table 1). Among 370 AA survivors (median age at blood draw for DNAm = 29.6 years, IQR = 23.8–37.0 years; 53.2% female), BMI was 12.6–58.9 kg/m^2^ (Table 1). The summary statistics of the weighted average levels of HDL, LDL, TG, and TC (including the number of survivors with multiple lipid measurements) before or after DNA sampling are shown in Table 1. Compared with survivors of EA, those of AA had lower mean of weighted average of TG as an exposure (81.2 vs. 125.3 mg/dL, *P* < 0.0001) and outcome (83.4 vs. 130.6, *P* < 0.0001), TC as an exposure (171.3 vs 179.2 mg/dL, *P*=0.02) and an outcome (163.8 vs. 182.3 mg/dL, *P* < 0.0001), and LDL as an outcome (95.0 vs. 106.1 mg/dL, *P* <0.0001). AA survivors also had higher mean of weighted average of HDL as an exposure (57.2 vs. 51.1 mg/dL, *P* < 0.0001). The correlations of weighted average levels of lipids before and after DNA sampling were shown in Additional file 1: Table S1. The percentage of survivors taking any lipid control medications before DNAm sampling was 8.04% in EA and 4.86% in AA. The median time and range between the DNAm and pre-lipid profiles are 1.6, 0.0–5.3, years for EA and 1.6, 0.5–5.1, years for AA, and the median time and range between DNAm and post-lipid profiles are 2.2, 0.0–5.5, years for EA, and 2.3, 0.1–16.6, years (Table 1).

**Table 1:** Characteristics of the SJLIFE study population

Characteristic	Survivors of European ancestry		Survivors of African ancestry		*P*^a^
	*n*	(%)	*n*	(%)	
Total	2052	(100.0)	370	(100.0)	
Sex					0.03
Male	1084	(52.8)	173	(46.8)	
Female	968	(47.2)	197	(53.2)	
Diagnosis					
Leukemia	699	(34.1)	77	(20.8)	< 0.0001
Acute lymphoblastic leukemia	644	(31.4)	67	(18.1)	
Acute myeloid leukemia	53	(2.6)	9	(2.4)	
Other leukemia	2	(0.1)	1	(0.3)	
Lymphoma	448	(21.8)	69	(18.6)	0.17
Hodgkin lymphoma	288	(14.0)	45	(12.2)	
Non-Hodgkin lymphoma	160	(7.8)	24	(6.5)	
Sarcoma	274	(13.4)	56	(15.1)	0.36
Ewing sarcoma	74	(3.6)	2	(0.5)	
Osteosarcoma	74	(3.6)	18	(4.9)	
Rhabdomyosarcoma	71	(3.5)	18	(4.9)	
Soft tissue sarcoma	55	(2.7)	18	(4.9)	
CNS tumors	231	(11.3)	45	(12.2)	0.61
Astrocytoma or glioma	93	(4.5)	18	(4.9)	
Medulloblastoma or PNET	56	(2.7)	10	(2.7)	
Ependymoma	26	(1.3)	5	(1.4)	
Other CNS tumors	56	(2.7)	12	(3.2)	
Embryonal	276	(13.5)	81	(21.9)	< 0.0001
Wilms tumor	134	(6.5)	44	(11.9)	
Neuroblastoma	107	(5.2)	14	(3.8)	
Germ cell tumor	35	(1.7)	23	(6.2)	
Other	124	(6.0)	42	(11.4)	0.0002
Retinoblastoma	45	(2.2)	21	(5.7)	
Hepatoblastoma	13	(0.6)	2	(0.5)	
Melanoma	12	(0.6)	2	(0.5)	
Carcinomas	24	(1.2)	14	(3.8)	
Others	30	(1.5)	3	(0.8)	
Chemotherapy					
Alkylating agent, classical	1194	(58.2)	202	(54.6)	0.20
Alkylating agent, heavy metal	239	(11.7)	63	(17.0)	0.004
Alkylating agent, nonclassical	67	(3.3)	12	(3.2)	0.98
Anthracyclines	1190	(58.0)	180	(48.6)	0.0008
Antimetabolites	1024	(49.9)	133	(35.9)	< 0.0001
Asparaginase enzymes	631	(30.8)	76	(20.5)	< 0.0001
Epipodophyllotoxins	709	(34.6)	108	(29.2)	0.04
Corticosteroids	965	(47.0)	122	(33.0)	< 0.0001
Vinca alkaloids	1482	(72.2)	236	(63.8)	0.0009
Radiation therapy, region exposed					
Brain	629	(30.7)	98	(26.5)	0.11
Chest	577	(28.1)	102	(27.6)	0.83
Abdominal	412	(20.1)	84	(22.7)	0.25
Pelvic	352	(17.2)	80	(21.6)	0.04
Tobacco smoking status					0.11
Never smoking	956	(46.6)	183	(49.5)	
Ever smoking	349	(17.0)	51	(13.8)	
Unknown	747	(36.4)	136	(36.8)	
Lipid control medication before DNA sampling					0.03
Never used	1887	(92.0)	352	(95.1)	
Ever used	165	(8.0)	18	(4.9)	
Lipid control medication after DNA sampling					0.06
Never used	1958	(95.4)	361	(97.6)	
Ever used	94	(4.6)	9	(2.4)	

Chronic health condition (Weighted average, mg/dL)	*n*	Mean ± SD	*n*	Mean ± SD	*P*^a^
Triglycerides, exposure	734	125.3 ± 92.9	117	81.2 ± 46.6	< 0.0001
Triglycerides, outcome	1126	130.6 ± 98.0	197	83.4 ± 47.8	< 0.0001
Total cholesterol, exposure	734	179.2 ± 34.9	117	171.3 ± 32.3	0.02
Total cholesterol, outcome	1124	182.3 ± 39.2	197	163.8 ± 48.0	< 0.0001
High-density lipoprotein, Exposure	734	51.1 ± 14.7	117	57.2 ± 14.2	< 0.0001
High-density lipoprotein, Outcome	1124	50.5 ± 16.3	197	52.3 ± 20.0	0.21
Low-density lipoprotein, Exposure	717	103.6 ± 29.6	116	98.3 ± 28.4	0.08
Low-density lipoprotein, Outcome	1092	106.1 ± 31.5	196	95.0 ± 35.3	< 0.0001
Body mass index, kg/m^2^	2051	28.5 ± 7.4	368	29.2 ± 8.0	0.13

	Median	Range	Median	Range	*P*^a^
Age at DNA sampling, years	32.3	18.0, 66.4	29.6	18.4, 65.1	< 0.0001
Median age of multiple lipid measurements, years^b^					
Exposure	30.0	14.6, 65.1	26.6	15.2, 56.6	0.03
Outcome	35.3	19.6, 67.8	34.0	20.0, 67.5	0.01
Time duration between DNA sampling and median age of multiple lipid measurements, years					
Exposure	1.6	0.0, 5.3	1.6	0.5, 5.1	0.41
Outcome	2.2	0.0, 5.5	2.3	0.1, 16.6	0.44

*CNS* central nervous system, *IQR* interquartile range, *PNET* primitive neuroectodermal tumor

^a^Chi-square test for categorical variables, Student’s *t*-test for continuous variables. Distribution differences for both age and time duration were assessed by Wilcoxon rank sum test

^b^The median age of multiple lipid measurements for each survivor was calculated, and the median across all survivors was subsequently derived

**Fig. 1:**
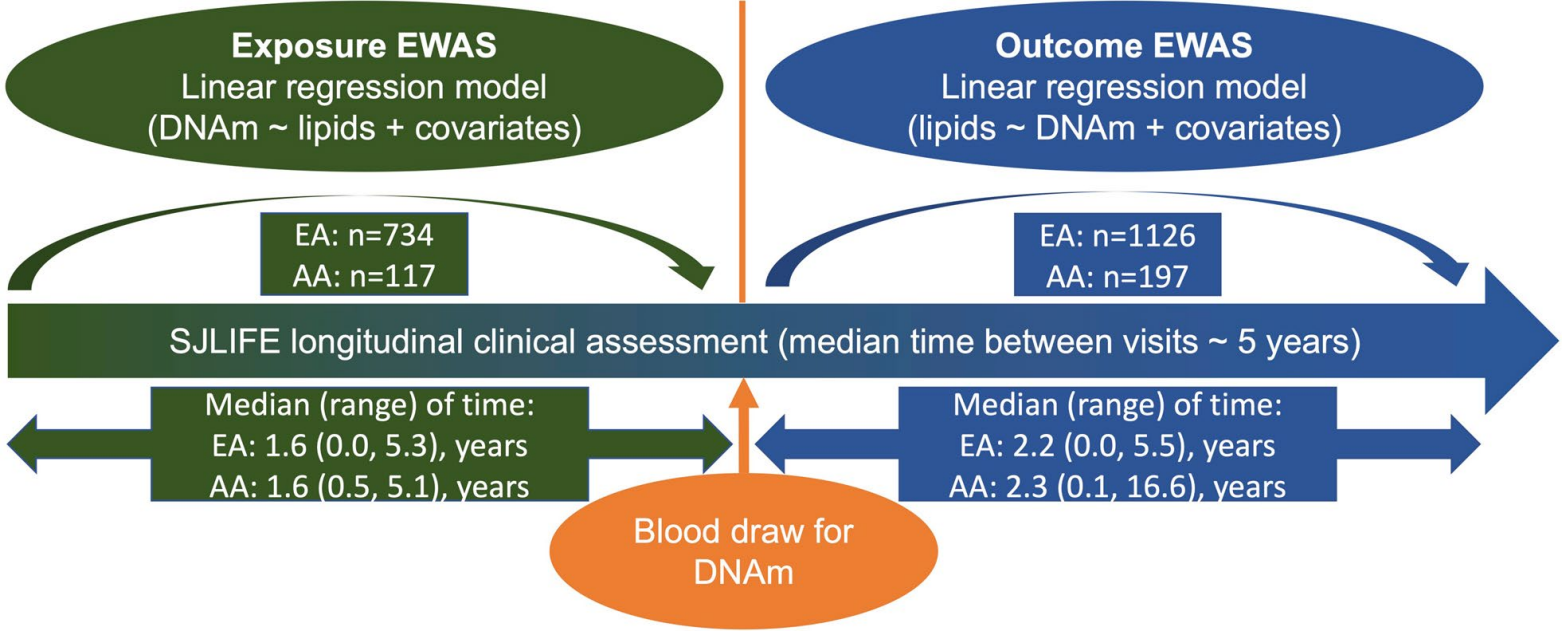
Schematic framework of study design. *EWAS* epigenome-wide association study, *DNAm* DNA methylation, *SJLIFE* St. Jude lifetime cohort study. Median (range) of time, the median/range of time between DNAm sampling age and median age of multiple lipid measurements

After quality control of DNAm data, a total of 689,414 CpGs were further advanced for EWAS. The associations between the DNAm level of each CpG and specific blood lipid level (HDL, LDL, TG, or TC) as an exposure or outcome were analyzed separately (Fig. 1). Quantile–quantile plots of each EWAS among survivors of EA and AA were shown in Additional file 1: Fig. S1 and Additional file 1: Fig. S2, respectively. EWAS of blood lipids among survivors of EA showed moderately low genomic inflation factors between 0.92 and 1.13 (Additional file 1: Fig. S1). EWAS of blood lipids among survivors of AA showed moderately low to high genomic inflation factors between 0.92 and 2.32 (Additional file 1: Fig. S2).

### CpG sites associated with blood lipids among survivors of European ancestry

**Fig. 2:**
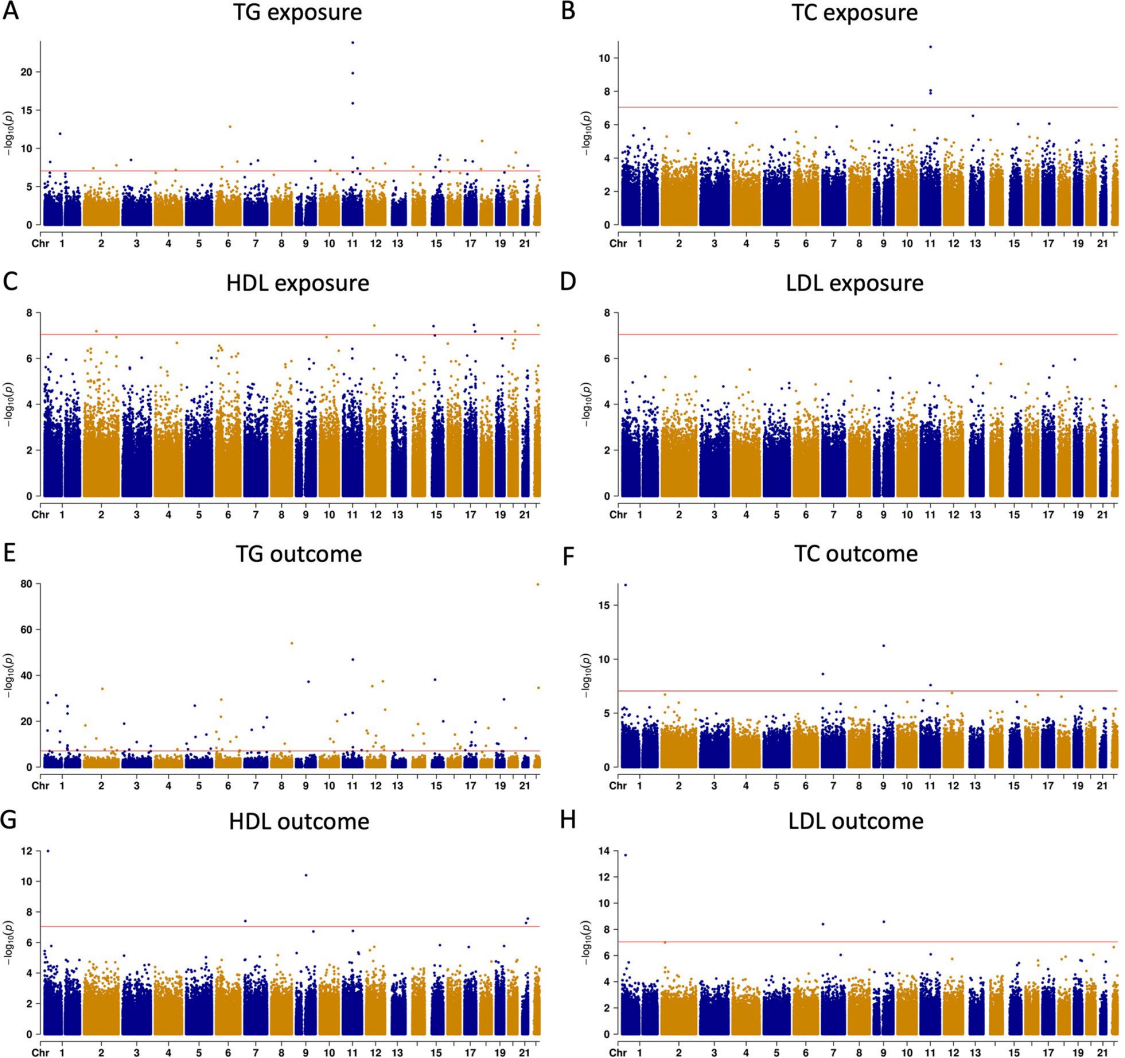
Manhattan plots of exposure and outcome EWAS of blood lipids among survivors of European ancestry in SJLIFE cohort. *EWAS* epigenome-wide association study, *SJLIFE* St. Jude lifetime cohort study, *HDL* high-density lipoprotein, *LDL* low-density lipoprotein, *TG* triglycerides, *TC* total cholesterol

The landscapes of the overall association results among survivors of EA were shown in Fig. 2. Seven, three, and 33 epigenome-wide significant blood lipid-associated CpGs were identified for HDL, TC, and TG, respectively, in the exposure EWAS (*P* < 9 × 10^−8^, Fig. 2A–C). No significant CpG achieved epigenome-wide significance in the LDL exposure EWAS among survivors of EA (*P* < 9 × 10^−8^, Fig. 2D). Detailed estimates for the association between each CpG and specific blood lipid level as an exposure were provided in Additional file 1: Table S2. Notably, a cluster of three CpGs (cg00574958, cg05325763, and cg17058475), mapped to the 5′UTR of the *CPT1A* gene, were common in the TG and TC exposure EWAS (Table 2 and Additional file 1: Fig. S3). Five, three, four, and 94 CpGs were significantly associated with HDL, LDL, TC, and TG, respectively, in the outcome EWAS (*P* < 9 × 10^−8^, Fig. 2E–H and Additional file 1: Table S3). Three CpGs were common across HDL, LDL, and TC outcome EWAS, including ch.1.829344F mapped to the 5′UTR region of the *SRPM1* gene, cg20935223 mapped to the 3′UTR region of the *CYTH3* gene, and cg21750129 mapped to the 3′UTR region of the *TRPM3* gene (Table 2 and Additional file 1: Fig. S3). No significant CpGs were common between blood lipid exposure and outcome EWAS (*P* < 9 × 10^−8^).

**Table 2:** Overlapping significant CpGs in exposure and outcome EWAS of blood lipid among survivors of European ancestry in SJLIFE cohort

CpG	chr_hg38	Start_hg38	Nearby gene gengebnegene	EWAS	Lipid	Effect	SE	*P*
cg00574958	11	68840153	*CPT1A*	Exposure	TC	− 2.25E−03	3.30E−04	2.15E−11
					TG	− 1.28E−03	1.20E−04	1.50E−24
cg05325763	11	68840250	*CPT1A*		TC	− 1.84E−03	3.20E−04	1.31E−08
					TG	− 1.13E−03	1.17E−04	1.48E−20
cg17058475	11	68840268	*CPT1A*		TC	− 2.05E−03	3.52E−04	8.81E−09
					TG	− 1.11E−03	1.31E−04	1.27E−16
ch.1.829344F	1	24657005	*SRRM1*	Outcome	HDL	6.39	0.89	1.03E−12
					LDL	17	2.19	2.18E−14
					TC	25.51	2.94	1.36E−17
cg20935223	7	6168606	*CYTH3*		HDL	8.30	1.50	3.96E−08
					LDL	22.27	3.75	3.99E−09
					TC	30.22	5.02	2.40E−09
cg21750129	9	71249778	*TRPM3*		HDL	7.03	1.05	3.97E−11
					LDL	15.86	2.64	2.63E−09
					TC	24.53	3.52	5.73E−12

*EWAS* epigenome-wide association study, *chr_hg38* chromosome in GRCh38/hg38, *Start_hg38* CpG start position in GRCh38/hg38, *SE* standard error, *HDL* high-density lipoprotein, *LDL* low-density lipoprotein, *TG* triglycerides, *TC* total cholesterol

### CpG sites associated with blood lipids among survivors of African ancestry

The overall landscape of CpG associations with blood lipid EWAS among survivors of AA were shown in the Additional file 1: Fig. S4. Six TG-associated CpGs were identified in the exposure EWAS (Additional file 1: Fig. S4A and Table S4), and five TG-associated CpGs, two TC-associated CpGs, one HDL-associated CpG, and one LDL-associated CpG were found in the outcome EWAS (Additional file 1: Fig. S4E–H and Table S4) (*P* < 9 × 10^−8^). No significant CpG was found in HDL, LDL, and TC exposure EWAS. Similarly, there was no significant CpGs common in exposure and outcome EWAS among survivors of AA (*P* < 9 × 10^−8^). In TG exposure EWAS, there were five significant CpGs mapping to nearby genes including cg26675329 and cg04747445 within 1500 bp upstream of the transcription start site of the *IL18RAP* gene and the *BBX* gene, respectively; cg05416955 and cg21376908 in the gene body of the *CARD9* gene and the *MSI2* gene, respectively; and cg16197879 in the 5’UTR region of the *CLDN14* gene (*P* < 9 × 10^−8^, Additional file 1: Table S4). In TC outcome EWAS, cg16411101 in the 5’UTR of the SSCP3 gene was significant in both LDL and TC outcome EWAS, and the other significant CpG was cg23724016 in the 3’UTR of the *CDHR5* gene (*P* < 9 × 10^−8^). In HDL outcome, one significant CpG cg14558275 is mapped to the *PIK3CG* gene. In TG outcome, cg05416345 and cg01111718 are in the gene body of the *IFFO2* gene and the *GRIA4* gene, respectively; cg04348872 and cg12686539 are in the first and second exon of the *ADCY3* gene and the *ZNF891* gene, respectively. We did not identify any overlapping significant CpG between survivors of EA and AA in SJLIFE cohort in any of the exposure or outcome EWAS of blood lipids (*P* < 9 × 10^−8^).

### Trans-ethnic meta-analysis

In the meta-analysis of blood lipid EWAS among EA and AA survivors, we identified 74 significant lipid-CpG associations (70 unique CpGs, *P* < 9 × 10^−8^). Specifically, four, one, and 33 significant CpGs were associated with HDL, TC, and TG exposures, respectively; and two, one, two, and 31 were associated with HDL, LDL, TC, and TG outcomes, respectively (*P* < 9 × 10^−8^, Additional file 1: Table S5). Among these significant lipid-CpG associations, twelve did not reach epigenome-wide significance level in either EWAS among survivors of EA or AA alone, including three for HDL exposure, three for TG exposure, and six for TG outcome (Table 3). All 12 had homogeneous effects with the same direction of association in survivors of EA and AA (*P*_het_ > 0.1, Table 3). Among the remaining 62 lipid-CpG associations that were significant in EWAS among survivors of EA or AA alone (*P* < 9 × 10^−8^), twenty-two had homogeneous effects with the same direction between survivors of EA and AA (*P*_het_ > 0.1), twenty-four had opposite directions of association with significant heterogeneity between survivors of AA and EA (*P*_het_ < 0.1), and the remaining 16 significant lipid-CpG associations either had the same direction of association but with significant heterogeneity between survivors of EA and AA (*P*_het_ < 0.1) or had the opposite direction of association but with homogenous effect (*P*_het_ > 0.1) (Additional file 1: Table S5).

**Table 3:** Additional significant CpGs identified in meta-analysis of blood lipids EWAS among survivors of European and African ancestry (*P* < 9 × 10^−8^)

EWAS	Lipid	CpG	chr_hg38	Start_hg38	Nearby genes	Population	Effect	SE	*P*	Directions (EA/AA)	*I*^2^	*P*_het_
Exposure	HDL	cg27324117	chr4	153793134	NA	EA	− 0.0025	0.0005	2.11E−07			
						AA	− 0.0016	0.0012	0.20			
						Meta	− 0.0024	0.0004	8.71E−08	−/−	0	0.4765
		cg02443467	chr10	47191407	NA	EA	− 0.0022	0.0004	1.17E−07			
						AA	− 0.0016	0.0010	0.09			
						Meta	− 0.0021	0.0004	2.30E−08	−/−	0	0.5916
		cg07373304	chr16	998982	*RP11-161M6.3*	EA	− 0.0021	0.0004	2.26E−07			
						AA	− 0.0044	0.0017	0.01			
						Meta	− 0.0023	0.0004	1.41E−08	−/−	36	0.2115
	TG	cg01082498	chr11	68840756	*CPT1A*	EA	− 0.0006	0.0001	1.24E−07			
						AA	− 0.0005	0.0007	0.48			
						Meta	− 0.0006	0.0001	7.23E−08	−/−	0	0.8322
		cg06004232	chr16	11293335	*RMI2*	EA	0.0003	6.09E−05	1.20E−07			
						AA	0.0007	0.0003	0.04			
						Meta	0.0003	0.0001	1.79E−08	+/+	5.7	0.3032
		cg11686214	chr19	58573616	*CENPBD1P1;MZF1;MZF1-AS1*	EA	0.0006	0.0001	1.45E−07			
						AA	0.0006	0.0006	0.24			
						Meta	0.0006	0.0001	5.20E−08	+/+	0	0.929
Outcome	TG	cg23550429	chr2	30797935	*CAPN13*	EA	− 40.3640	20.2715	0.05			
						AA	− 14.9963	2.8360	3.37E−07			
						Meta	− 15.4833	2.8087	3.54E−08	−/−	34.9	0.2152
		cg03260858	chr3	49103337	*QARS*	EA	20.1158	13.6051	0.14			
						AA	38.5596	6.9392	9.14E−08			
						Meta	34.7521	6.1816	1.89E−08	+/+	31.4	0.2272
		cg20601481	chr11	67288314	*ANKRD13D*	EA	− 27.9673	10.8875	0.01			
						AA	− 37.6708	7.6983	2.10E−06			
						Meta	− 34.4364	6.2857	4.29E−08	−/−	0	0.4668
		cg19590858	chr14	34540645	*EAPP;RP11-671J11.5*	EA	− 31.9752	9.1836	5.17E−04			
						AA	− 27.2994	6.6324	5.73E−05			
						Meta	− 28.9022	5.3768	7.64E−08	−/−	0	0.6798
		cg20433103	chr17	63941062	*SCN4A*	EA	43.8451	13.1675	8.98E−04			
						AA	36.7039	8.0731	9.67E−06			
						Meta	38.6549	6.8825	1.95E−08	+/+	0	0.6438
		cg07439166	chr19	35902502	*AD000864.6;HCST;NFKBID*	EA	− 51.5564	15.2318	7.37E−04			
						AA	− 34.0310	7.4137	8.01E−06			
						Meta	− 37.3876	6.666	2.04E−08	−/−	6.6	0.3009

*chr_hg38* chromosome in GRCh38/hg38, *Start_hg38* CpG start position in GRCh38/hg38, *HDL* high-density lipoprotein, *LDL* low-density lipoprotein, *TG* triglycerides, *TC* total cholesterol, *EA* survivors of European ancestry, *AA* survivors of African ancestry

### Association between DNAm levels of lipid–associated CpGs and gene expression

For each of the blood-lipid associated CpGs, we estimated the linear association between DNAm levels and gene expression levels (adjusting for DNA/RNA sampling age and sex). Among the nearby genes of the significant lipid-associated CpGs among EA, there were no count data (number of reads from RNA sequencing) for 52 genes. Of the remaining 56 CpG-gene pairs, there were ten CpG-gene pairs with significant (FDR < 0.05) associations between the DNAm level of lipid-associated CpGs and gene expression of their nearby genes, including *HDAC7* (cg01620154), *AXIN2* (cg23475474), *ECE1* (cg01758046), *TRERF1* (cg07507418), *TNRC6B* (cg00543524), *MICU1* (cg08641767), *NUDCD3* (cg01507280), *NLN* (cg01710244), *AKAP1* (cg18807499), and *LRP5* (cg24040155) among EA (Table 4). Most of the estimated effects of these CpGs were negative (i.e., increased methylation associated with decreased gene expression), except for *ECE1* (cg01758046), *NUDCD3* (cg01507280), and *NLN* (cg01710244). Among the 12 annotated genes of significant lipid-associated CpGs among AA survivors (Additional file 1: Table S4), *nine* gene had no count data (i.e., number of reads from RNA sequencing). Of the remaining three CpG-gene pairs (*PIK3CG*-cg14558275, *BBX*-cg04747445, and *IFFO2*-cg05416345), there was no significant association between DNAm levels of lipid-associated CpGs and the gene expression among AA.

**Table 4:** Significant associations between significant CpGs identified in blood lipid (exposure/outcome) EWAS among survivors of European ancestry and the expression of their nearby genes (FDR < 0.05)

CpG	Gene Ensembl ID	Nearby gene	Effect	SE	*P*	FDR
cg01620154	ENSG00000061273	*HDAC7*	− 3.68	0.63	4.43E−08	1.24E−06
cg23475474	ENSG00000168646	*AXIN2*	− 4.30	0.72	2.54E−08	1.24E−06
cg01758046	ENSG00000117298	*ECE1*	2.28	0.56	8.54E−05	1.34E−03
cg07507418	ENSG00000124496	*TRERF1*	− 3.49	0.87	9.55E−05	1.34E−03
cg00543524	ENSG00000100354	*TNRC6B*	− 1.49	0.39	1.69E−04	1.89E−03
cg08641767	ENSG00000107745	*MICU1*	− 1.74	0.47	2.75E−04	2.57E−03
cg01507280	ENSG00000015676	*NUDCD3*	3.13	0.92	8.48E−04	6.78E−03
cg01710244	ENSG00000123213	*NLN*	5.50	1.69	1.45E−03	1.01E−02
cg18807499	ENSG00000121057	*AKAP1*	− 3.88	1.45	8.27E−03	4.63E−02
cg24040155	ENSG00000162337	*LRP5*	− 6.12	2.28	8.09E−03	4.63E−02

*SE* standard error, *FDR* false discovery rate

### Cross-reference with the EWAS Catalog

By comparing our findings with previously reported blood lipid-associated CpGs in the general population from the EWAS Catalog, only four overlapping CpGs were identified among EA survivors, including cg00574958 (associated with TG and TC exposure), cg09737197 (associated with TG exposure), and cg17058475 (associated with TG and TC exposure) in *CPT1A*, and cg03725309 (associated with TG exposure) in *SARS* (Additional file 1: Tables S6 and S7), and none among AA survivors. Among the remaining 136 novel lipid-associated CpGs in childhood cancer survivors of EA, 26 were mapped to 23 genes that have been previously reported as lipid-associated (Additional file 1: Table S8). Among 12 additional blood lipid-associated CpGs identified in the meta-EWAS, five CpGs were reported to be associated with other traits (e.g., sex, age, Schizophrenia, and ADHD (attention-deficit and hyperactivity disorder)) but only cg01082498 (in the 5’UTR region of the *CPT1A* gene) was associated with blood lipid level in the EWAS Catalog (Additional file 1: Table S9).

## Discussion

Genetic and epigenetic (specifically, DNAm) studies have identified numerous genetic variants or CpG sites that are associated with blood lipids in the general population, hence at least 2572 genes have been implicated in lipid metabolism (Additional file 1: Fig. S5) [17, 18]. We conducted the first EWAS of blood lipids among childhood cancer survivors, including EA and AA survivors from the SJLIFE cohort. Among EA survivors, we identified 149 (140 unique CpGs) significant associations with blood lipid levels; 136 of these were novel findings. Among AA survivors, we found 14 novel significant blood lipid-associated CpGs. There was no overlapping CpGs between EA and AA survivors. A majority of these findings are unique to the survivor population, which may be attributable to childhood cancer diagnoses and/or treatments. For example, two TG exposure associated CpGs, cg24327132 and cg19120513, were associated with chest-RT and abdominal-RT [6].

In the meta-EWAS, twenty-four CpGs had opposite direction of association with significant heterogeneity between EA and AA survivors (*P*_het_ < 0.1), suggesting substantial disparity in lipid-associated CpGs between the two ancestral groups. Meta-EWAS yielded eight additional epigenome-wide significant CpGs with heterogeneity in effect size between EA and AA survivors (*P*_het_ < 0.1). However, future replication in EA or AA alone with independent data set is warranted to validate such findings.

TG outcome EWAS yielded the greatest number of significant CpGs among EA, with multiple novel lipid-associated CpGs mapped to the same nearby genes, including *CDK5RAP3*, *FCGR2B*, *HSPA6*, and *HSPA7*. *CDK5RAP3,* known to play important roles in liver development and hepatic function. Previous research showed that hepatocyte-specific *Cdk5rap3* knockout mice suffered post-weaning lethality because of impaired lipid metabolism and serious hypoglycemia [19]. *FCGR2B* gene encodes FcγRIIb, with a novel role in CD11c^+^ cells in modulating serum cholesterol and triglyceride levels and maintaining liver cholesterol homeostasis [20]. *HSPA6* and *HSPA7* are family members of the *HSP70* proteins, which are abundantly present in cancer and play crucial roles in cancer development, progression, and metastasis, clinically resulting in diverse outcomes for patient survival [21]. Moreover, cg20935223 was significantly associated with multiple lipid traits in the outcome EWAS and mapped to *CYTH3* gene. *CYTH3* gene encodes Cytohesin-3, which is essential for insulin receptor signaling and body fat regulation via lipid excretion [22]. Among novel genes (i.e., not genes with nearby lipid-associated CpGs in EWAS Catalog), four of them were reported as high-confidence genes that play a role in lipid levels, including *LPIN2* (near cg07616376 associated with TG exposure among EA), *SCARB1* (near cg08458758 associated with TG outcome among EA), *MSI2* (near cg21376908 associated with TG exposure among AA), and *SSBP3* (near cg16411101 associated with LDL and TC outcome among AA) [23].

We integrated gene expression levels from RNA sequencing to further characterize the associations between DNAm and blood lipid levels, which strengthened this study. For example, we demonstrated that ten blood lipid-associated CpGs were associated with levels of expression of the annotated genes, in which seven were inversely associated.

However, it is important to note that there are several limitations in this study. First, although we innovatively designed both exposure and outcome EWAS based on our longitudinal follow-up study, the cross-sectional nature of the data prevented us from disentangling the complex interplay between DNAm and blood lipid levels. Nevertheless, we demonstrated potential regulation of gene expression as plausible mechanisms for DNAm alterations by performing RNA-sequencing analysis. Second, the sample size of survivors of AA was limited, that led to the limited power and the exploratory nature of the AA EWAS (i.e., some findings might be identified by chance). However, differences between EA and AA populations as determined by their genetic ancestry were observed with no overlapping blood lipid levels associated CpG between survivors of EA and those of AA. To further validate the findings, a larger sample size of AA survivors is warranted in the future. Previous methodological work suggested that more than 1,000 subjects are required to achieve 80% power for detection of differential DNAm at nominal genome-wide significance with an odds ratio of 1.15 [24]. Third, we obtained DNAm data at only one time point. In the outcome EWAS, all the blood lipid levels were measured after blood draw for DNAm, so the DNAm may be predictive of blood lipid levels. However, to better assess and interpret the changes of blood lipids level in exposure EWAS, longitudinal DNAm measurement (ideally, after the first blood lipid level measurement) is required to correlate changes of DNAm between two time points with changes in blood lipid levels. Fourth, the follow-up of our cohort is limited and still-ongoing, so there was large proportion of missing data in the analytic setting of bi-directional association between DNAm and lipid levels which requires multiple clinical assessments of lipid levels. Lastly, we did not consider cell type-specific DNAm in the current work. Recent research identified that DNAm variation in diseases, such as type 1 diabetes, can be cell type-specific [25]. Therefore, in the future, we may deconvolute bulk DNAm measured in blood leukocytes into cell type–specific quantities and analyze the DNAm associations of each specific cell type.

## Conclusions

Our findings demonstrated distinct DNAm signatures associated with blood lipid levels in EA and AA survivors, and that an additional set of genes may be implicated in lipid metabolism in the survivor population compared to the general population. Further longitudinal studies are warranted to replicate and validate DNAm biomarkers for blood lipid levels and other CHCs to facilitate the clinical translation for improved survivorship care.

## Methods

### Study population

SJLIFE is a retrospectively-constructed cohort with periodic evaluations of survivors beyond 5-years from childhood cancer diagnosis who were treated at St. Jude Children’s Research Hospital. The details of SJLIFE cohort study have been previously described [15, 16, 26]. Participants complete questionnaires assessing demographic and clinical factors, and receive comprehensive medical and laboratory assessments at each visit to determine health conditions. In this study, a total of 2,052 survivors of EA and 370 survivors of AA, with genome-wide DNAm profiling data, were included [27]. The ancestry for each survivor was determined using genotypes derived from whole-genome sequencing and population admixture analysis as previously described [28]. Primary childhood cancer diagnoses, exposure to chemotherapeutic agents and region-specific radiation dosimetry was obtained from medical records. All SJLIFE survivors completed at least one comprehensive clinical assessment that included a battery of laboratory tests including blood lipid measurement (HDL, LDL, TC, and TG) [26]. The blood lipid levels measured before blood sampling for DNAm were used for exposure EWAS and the blood lipid levels measured after were used in outcome EWAS (Fig. 1). Weighted average was calculated if there were multiple measurements, and time intervals between two consecutive measurements were used as weights. We excluded lipid measurements without fasting. Samples with only one lipid measurements (coinciding with the time point for the blood draw for DNAm) were excluded to ensure that our exposure and outcome EWAS examined the temporal association between DNAm and blood lipid levels. All participants provided written informed consent, with institutional review board approval at St. Jude Children’s Research Hospital.

### DNAm profiling and data processing

Illumina Infinium® MethylationEPIC BeadChip array including 850K CpG sites was used to generate genome-wide DNAm profiling on DNA derived from peripheral blood mononuclear cells (PBMC) collected at each follow-up visit for SJLIFE survivors. Details about laboratory experimental processes, array scanning, and DNAm bioinformatics data analysis were previously described by Song et al. [6].

### Genotyping based on whole-genome sequencing (WGS)

Genotyping was based on whole-genome sequencing data of blood derived DNA from 4402 SJLIFE survivors as previously described [29, 30]. Details about data processing, genotyping calling as well as additional genotype quality control criteria and procedures were previously described in Dong et al. [31].

### Epigenome-wide association analysis

Bidirectional EWAS was conducted using a multivariable linear regression to test the association of DNAm levels at each CpG (M-value, continuous variable) with blood lipid levels (continuous variable). We performed principal components analysis of methylation levels of all CpG sites to quantify potential batch effects in the DNAm data. The top four principal components were determined by the change rate of eigenvalues [6] and were included as covariates in the regression model. We also performed principal components analysis of genotypes derived from WGS to quantify the population substructure in EA and AA survivors. The top four principal components were determined by the change rate of eigenvalues and were included as covariates in the regression model. In the exposure EWAS, a multivariable linear regression model was used with lipid level (weighted average was calculated if there were multiple measurements, and time intervals between two consecutive measurements were used as weights) prior to DNA sampling as an independent variable and DNAm as a dependent variable, adjusting for sex, age at DNA sampling, leukocyte subtype proportions, top four significant genetic principal components, top four methylation principal components, cancer treatments, median age of lipid measurement, BMI, cigarette smoking, and lipid lowering medicine use. All these covariates were potential confounding factors for DNAm level of each CpG, and hence were considered in the exposure EWAS. Cancer treatments included chemotherapy and radiation therapy within 5 years from primary childhood cancer diagnosis. The chemotherapy agents included classical alkylating agent, anthracyclines, corticosteroids, vinca alkaloids, asparaginase enzymes, antimetabolites, and epipodophyllotoxins. The region-specific RT included brain-RT, chest-RT, abdomen-RT, and pelvis-RT. For smoking status as a categorical variable, we included three levels (“never”, “ever”, and “unknown”) in the model. BMI was measured at the same time as DNAm sampling. CpGassoc R package was used for the exposure EWAS analyses [32]. In the outcome EWAS, a multivariable linear regression model was used for DNAm (age-, sex-, cell-type-, genotype principal components-, and methylation principal components- adjusted) as an independent variable and lipid level (weighted average was calculated if there were multiple measurements, and time intervals between two consecutive measurements were used as weights) after DNA sampling as a dependent variable. For EA survivors, a base model without DNAm level but including the complete set of covariate (i.e., sex, cancer treatments, median age of lipid measurement, BMI, smoking, lipid lowering medicine use, lipid level measured at DNA sampling, age at DNA sampling, and polygenic risk score for specific lipid level (in EA only) was fitted. Cancer treatment exposures that were not statistically significant (*P* > 0.05) in the base model were subsequently excluded. In the final model, DNAm level of each CpG was added for the EWAS analysis. For AA survivors, considering the smaller sample size and potential overfitting, a similar but slightly different variable selection approach was taken by additionally excluding BMI, smoking status, and lipid lowering medicine use if any of these was not statistically significant (*P* > 0.05) in the base model. Polygenic risk score for specific lipid level was constructed by following the same approach described previously [28] for EA survivors. Custom R code was used for the outcome EWAS analyses. A *P* value less than 9 × 10^−8^ was deemed as epigenome-wide significance level corresponding to 5% family-wise error [33].

### RNA-sequence profiling and data processing

RNA was extracted from the same PBMC used for DNA methylation profiling. Details of library construction, sequencing, and data processing were described previously [27]. Briefly, paired-end 100 cycle sequencing was performed on a NovaSeq 6000 (Illumina). After quality control procedures, raw reads from the fastq files were aligned to the GRCh38.p13 version (v31) of the reference human genome from GENCODE through the automated internal pipeline [34]. The generated bam files were sorted and used to build an index using Samtools (version 1.9) [35] then used as inputs for counting reads using htseq-count [36] with GENCODE v31 gene annotation gtf file.

A total of 165 samples of RNA-seq data (135 EA survivors and 30 AA survivors) were available for further analysis. After removing transcripts with mean read counts across all 165 samples less than 10, a total of 12,882 genes were determined to be expressed in PBMC. Transcripts per million (TPM) [37] were calculated and transformed in the form of log_2_(TPM + 0.01). The function normalizeQuantiles in the limma package [38] in R (version 3.6.1) was used for quantile normalization [39] of the log-transformed values before further downstream analyses.

### Expression quantitative trait methylation

We used the Infinium® MethylationEPIC BeadChip array annotations (v1.0 B5) provided by Illumina (https://webdata.illumina.com/downloads/productfiles/methylationEPIC/infinium-methylationepic-v-1-0-b5-manifest-file-csv.zip) to map CpGs to their annotated genes. For 135 RNA-seq samples from EA survivors (out of 165 in total), the normalized expression values of the nearest genes of each lipid-associated CpGs were extracted to fit a linear regression against the DNAm levels of each CpG.

### Additional statistical and bioinformatic analyses

Quantile–Quantile plots were generated from *P*-values in each EWAS using the R lattice package. GenABEL R package [40] was used to estimate genomic inflation factor (i.e., lambda). We searched lipid-associated CpGs and nearby genes identified in our study and compared with those previously reported from the EWAS Catalog [17].

## Supplementary Information

**Additional file 1. **Supplementary Information.

## Data Availability

The DNA methylation data is accessible through the St. Jude Cloud (https://stjude.cloud).

## Abbreviations

AA: African ancestry
CpG: 5′-Cytosine-phosphate-guanine-3′
CHCs: Chronic health conditions
DNAm: DNA methylation
EA: European ancestry
HDL: High-density lipoprotein
LDL: Low-density lipoprotein
RT: Radiotherapy
SJLIFE: St. Jude lifetime cohort study
EA: European ancestry
TG: Triglycerides
TC: Total cholesterol
