# Supplementary material for: Distinct DNA methylation signatures associated with blood lipids as exposures or outcomes among survivors of childhood cancer: a report from the St. Jude lifetime cohort
Source: Clin Epigenetics. 2023 Feb 28;15:32. doi: 10.1186/s13148-023-01447-3 (PMC9976538; doi:10.1186/s13148-023-01447-3)
Supplement: Supplementary file 1 — Additional file 1. Supplementary Information. [file 13148_2023_1447_MOESM1_ESM.docx]

Additional file 1: Table S1. The correlation of weighted average levels of lipids before and after DNA sampling based on the overlapping survivors.

|  | Survivors of European ancestry | | |  | Survivors of African ancestry | | |
| --- | --- | --- | --- | --- | --- | --- | --- |
|  | N | Correlation | P |  | N | Correlation | P |
| Triglycerides | 446 | 0.66 | <0.001 |  | 60 | 0.95 | <0.001 |
| Total cholesterol | 444 | 0.80 | <0.001 |  | 60 | 0.91 | <0.001 |
| High-density lipoprotein | 444 | 0.92 | <0.001 |  | 60 | 0.93 | <0.001 |
| Low-density lipoprotein | 421 | 0.84 | <0.001 |  | 59 | 0.90 | <0.001 |

Additional file 1: Table S2. Significant CpGs in exposure EWAS of blood lipid among survivors of European ancestry in SJLIFE cohort (*P*<9x10^-8^).

| EWAS | CpG | chr | start | end | Nearby Gene | Effect size | (SE) | *P* | UCSC CpG Island | Regulatory Feature group |
| --- | --- | --- | --- | --- | --- | --- | --- | --- | --- | --- |
| HDL exposure | cg23475474 | 17 | 65531805 | 65531807 | *AXIN2* | -2.40E-03 | 4.30E-04 | 3.46E-08 |  |  |
|  | cg00543524 | 22 | 40210132 | 40210134 | *TNRC6B* | -2.51E-03 | 4.51E-04 | 3.60E-08 |  |  |
|  | cg16500637 | 12 | 56291885 | 56291887 | *CS;RP11-977G19.10* | 2.87E-03 | 5.15E-04 | 3.67E-08 |  |  |
|  | cg12300603 | 15 | 31308446 | 31308448 | *NA* | -2.00E-03 | 3.60E-04 | 3.89E-08 |  |  |
|  | cg03954453 | 2 | 86035563 | 86035565 | *POLR1A* | -2.21E-03 | 4.04E-04 | 6.51E-08 |  |  |
|  | cg07112491 | 17 | 73737091 | 73737093 | *CTD-2532D12.4* | -1.75E-03 | 3.20E-04 | 6.69E-08 |  |  |
|  | cg14543017 | 20 | 45944371 | 45944373 | *PCIF1* | 2.55E-03 | 4.67E-04 | 6.70E-08 | N shore |  |
| TC exposure | cg00574958 | 11 | 68840153 | 68840155 | *CPT1A* | -2.25E-03 | 3.30E-04 | 2.15E-11 |  |  |
|  | cg17058475 | 11 | 68840268 | 68840270 | *CPT1A* | -2.05E-03 | 3.52E-04 | 8.81E-09 |  |  |
|  | cg05325763 | 11 | 68840250 | 68840252 | *CPT1A* | -1.84E-03 | 3.20E-04 | 1.31E-08 |  |  |
| TG exposure | cg00574958 | 11 | 68840153 | 68840155 | *CPT1A* | -1.28E-03 | 1.20E-04 | 1.50E-24 |  |  |
|  | cg05325763 | 11 | 68840250 | 68840252 | *CPT1A* | -1.13E-03 | 1.17E-04 | 1.48E-20 |  |  |
|  | cg17058475 | 11 | 68840268 | 68840270 | *CPT1A* | -1.11E-03 | 1.31E-04 | 1.27E-16 |  |  |
|  | ch.6.98859407F | 6 | 98304809 | 98304810 | *RP11-436D23.1* | 7.95E-04 | 1.06E-04 | 1.47E-13 |  |  |
|  | cg03725309 | 1 | 109214962 | 109214964 | *SARS* | -5.76E-04 | 7.97E-05 | 1.23E-12 |  |  |
|  | cg10567810 | 18 | 11149068 | 11149070 | *PIEZO2* | 7.38E-04 | 1.07E-04 | 1.07E-11 |  |  |
|  | cg09720768 | 20 | 50645405 | 50645407 | *RIPOR3;RP4-530I15.6* | -4.26E-04 | 6.70E-05 | 3.58E-10 |  |  |
|  | cg25600227 | 15 | 78193555 | 78193557 | *ACSBG1* | -4.43E-04 | 7.13E-05 | 8.64E-10 |  |  |
|  | cg09737197 | 11 | 68840206 | 68840208 | *CPT1A* | -6.41E-04 | 1.05E-04 | 1.60E-09 |  |  |
|  | cg24327132 | 15 | 72228290 | 72228292 | *PKM* | -4.53E-04 | 7.53E-05 | 2.79E-09 | N shore |  |
|  | cg10886794 | 16 | 290026 | 290028 | *AXIN1* | -3.79E-04 | 6.33E-05 | 3.22E-09 | N shore |  |
|  | cg02658330 | 3 | 58667865 | 58667867 | *FAM3D* | -4.13E-04 | 6.90E-05 | 3.31E-09 |  |  |
|  | cg23623659 | 17 | 5022179 | 5022181 | *KIF1C* | -9.49E-04 | 1.59E-04 | 3.75E-09 |  |  |
|  | cg12397924 | 7 | 94657873 | 94657875 | *PEG10* | -5.09E-04 | 8.53E-05 | 3.88E-09 | S shore | Promoter |
|  | cg03360355 | 9 | 135352663 | 135352665 | *RP11-555H7.2* | -2.74E-04 | 4.61E-05 | 4.75E-09 |  |  |
|  | cg18807499 | 17 | 57096382 | 57096384 | *AKAP1;RP11-166P13.4* | -3.52E-04 | 5.95E-05 | 5.16E-09 |  |  |
|  | cg03982376 | 6 | 149485522 | 149485524 | *ZC3H12D* | -4.80E-04 | 8.13E-05 | 5.41E-09 |  |  |
|  | ch.1.1262625R | 1 | 39747240 | 39747241 | *PPIE* | 6.49E-04 | 1.10E-04 | 6.03E-09 |  |  |
|  | cg10417863 | 12 | 132780368 | 132780370 | *GOLGA3* | -3.32E-04 | 5.72E-05 | 9.41E-09 |  |  |
|  | cg01507280 | 7 | 44472076 | 44472078 | *NUDCD3* | -4.97E-04 | 8.59E-05 | 1.11E-08 |  |  |
|  | cg17250680 | 2 | 226981934 | 226981936 | *RHBDD1* | 3.14E-04 | 5.50E-05 | 1.66E-08 |  |  |
|  | cg16740586 | 21 | 42235808 | 42235810 | *ABCG1* | 4.52E-04 | 7.93E-05 | 1.76E-08 | S shore |  |
|  | cg09097291 | 20 | 1170871 | 1170873 | *PSMF1* | -5.11E-04 | 9.00E-05 | 2.04E-08 |  |  |
|  | cg05032656 | 14 | 23729804 | 23729806 | *RP11-388E23.2* | -5.13E-04 | 9.11E-05 | 2.59E-08 |  |  |
|  | cg07507418 | 6 | 42404545 | 42404547 | *TRERF1* | -2.66E-04 | 4.73E-05 | 2.61E-08 |  |  |
|  | cg12604154 | 15 | 45390179 | 45390181 | *GATM* | -3.37E-04 | 6.01E-05 | 2.79E-08 |  |  |
|  | cg05287486 | 20 | 35257395 | 35257397 | *MMP24;MMP24OS* | -4.18E-04 | 7.49E-05 | 3.37E-08 |  |  |
|  | cg01620154 | 12 | 47798315 | 47798317 | *HDAC7* | 3.64E-04 | 6.55E-05 | 3.80E-08 |  |  |
|  | cg08395475 | 2 | 65277850 | 65277852 | *NA* | -4.34E-04 | 7.81E-05 | 4.01E-08 |  |  |
|  | cg19120513 | 11 | 102318571 | 102318573 | *BIRC3* | -2.84E-04 | 5.12E-05 | 4.28E-08 |  |  |
|  | cg07616376 | 18 | 2980516 | 2980518 | *LPIN2;RP11-737O24.1* | -6.58E-04 | 1.19E-04 | 5.07E-08 |  |  |
|  | cg25972069 | 4 | 145881853 | 145881855 | *ZNF827* | 2.41E-04 | 4.42E-05 | 6.66E-08 |  |  |
|  | cg12726743 | 10 | 73875089 | 73875091 | *CAMK2G* | -3.87E-04 | 7.13E-05 | 7.56E-08 |  |  |

Abbreviations: EWAS, epigenome-wide association study; chr, chromosome; start, CpG start position in GRCh38/hg38; end, CpG end position in GRCh38/hg38; SE, standard error; HDL, high-density lipoprotein; TG, triglycerides; TC, total cholesterol.

Additional file 1: Table S3. Significant CpGs in outcome EWAS of blood lipid among survivors of European ancestry in SJLIFE cohort (*P*<9x10^-8^).

| EWAS | CpG | chr | start | end | Nearby Gene | Effect size | (SE) | *P* | UCSC CpG Island | Regulatory Feature group |
| --- | --- | --- | --- | --- | --- | --- | --- | --- | --- | --- |
| HDL outcome | ch.1.829344F | 1 | 24657005 | 24657006 | *SRRM1* | 6.39 | 0.89 | 1.03E-12 |  |  |
|  | cg21750129 | 9 | 71249778 | 71249780 | *TRPM3* | 7.03 | 1.05 | 3.97E-11 |  |  |
|  | cg11818343 | 21 | 44398007 | 44398009 | *TRPM2* | 7.22 | 1.29 | 2.73E-08 |  |  |
|  | cg20935223 | 7 | 6168606 | 6168608 | *CYTH3* | 8.30 | 1.50 | 3.96E-08 |  |  |
|  | cg03182645 | 21 | 32021884 | 32021886 | *HUNK;HUNK-AS1* | -6.27 | 1.14 | 5.26E-08 |  |  |
| LDL outcome | ch.1.829344F | 1 | 24657005 | 24657006 | *SRRM1* | 17.00 | 2.19 | 2.18E-14 |  |  |
|  | cg21750129 | 9 | 71249778 | 71249780 | *TRPM3* | 15.86 | 2.64 | 2.63E-09 |  |  |
|  | cg20935223 | 7 | 6168606 | 6168608 | *CYTH3* | 22.27 | 3.75 | 3.99E-09 |  |  |
| TC outcome | ch.1.829344F | 1 | 24657005 | 24657006 | *SRRM1* | 25.51 | 2.94 | 1.36E-17 |  |  |
|  | cg21750129 | 9 | 71249778 | 71249780 | *TRPM3* | 24.53 | 3.52 | 5.73E-12 |  |  |
|  | cg20935223 | 7 | 6168606 | 6168608 | *CYTH3* | 30.22 | 5.02 | 2.40E-09 |  |  |
|  | cg26253297 | 11 | 70534766 | 70534768 | *SHANK2* | -24.85 | 4.43 | 2.56E-08 |  |  |
| TG outcome | cg18452324 | 22 | 37486358 | 37486360 | *MFNG* | 133.16 | 6.45 | 2.38E-80 |  |  |
|  | cg15295857 | 8 | 144448140 | 144448142 | *NA* | -286.36 | 17.39 | 1.04E-54 | N shore |  |
|  | cg08657939 | 11 | 69480238 | 69480240 | *AP000439.3* | -227.07 | 14.93 | 1.31E-47 |  |  |
|  | cg20703271 | 15 | 41822236 | 41822238 | *MAPKBP1* | -174.42 | 12.87 | 7.64E-39 |  |  |
|  | cg25089643 | 12 | 116336157 | 116336159 | *NA* | 158.23 | 11.80 | 4.10E-38 |  |  |
|  | cg05453148 | 9 | 87607479 | 87607481 | *DAPK1* | -136.48 | 10.21 | 6.29E-38 |  |  |
|  | cg11570494 | 12 | 42235171 | 42235173 | *YAF2* | -130.21 | 10.03 | 5.13E-36 | N shelf |  |
|  | cg10769317 | 22 | 42344268 | 42344270 | *TCF20* | 73.03 | 5.69 | 2.74E-35 |  |  |
|  | cg00656366 | 2 | 128550034 | 128550036 | *NA* | -196.63 | 15.43 | 7.84E-35 |  |  |
|  | cg13695997 | 1 | 81525284 | 81525286 | *ADGRL2;RP5-837I24.2* | -125.82 | 10.34 | 4.54E-32 |  |  |
|  | cg26471866 | 19 | 55542146 | 55542148 | *SBK3* | -151.89 | 12.90 | 2.96E-30 |  |  |
|  | cg18435125 | 6 | 36721521 | 36721523 | *RAB44* | -127.67 | 10.86 | 3.84E-30 |  |  |
|  | cg11505417 | 1 | 22639557 | 22639559 | *C1QA* | 57.49 | 5.02 | 9.51E-29 |  |  |
|  | cg01233571 | 5 | 60651660 | 60651662 | *DEPDC1B* | -97.72 | 8.76 | 1.72E-27 |  |  |
|  | cg26156167 | 1 | 161612860 | 161612862 | *FCGR2B* | 77.56 | 6.97 | 2.45E-27 |  |  |
|  | cg17405122 | 1 | 161525061 | 161525063 | *HSPA6;RP11-25K21.6* | -145.84 | 13.15 | 3.46E-27 |  |  |
|  | cg20054394 | 12 | 132361751 | 132361753 | *RP13-895J2.11* | -117.76 | 10.94 | 9.32E-26 |  |  |
|  | cg24040155 | 11 | 68433266 | 68433268 | *LRP5* | -99.40 | 9.53 | 2.38E-24 |  |  |
|  | cg13495918 | 1 | 161605533 | 161605535 | *FCGR2B;FCGR2C;HSPA7* | -75.96 | 7.33 | 4.54E-24 | N shore |  |
|  | cg16183736 | 11 | 17021687 | 17021689 | *OR7E14P* | -88.58 | 8.64 | 1.29E-23 |  |  |
|  | cg22467444 | 6 | 33930874 | 33930876 | *NA* | -144.88 | 14.47 | 1.17E-22 |  |  |
|  | cg17960556 | 7 | 157044322 | 157044324 | *NA* | -98.09 | 9.87 | 2.28E-22 |  |  |
|  | cg26727817 | 10 | 121617326 | 121617328 | *RP11-62L18.3* | -83.69 | 8.78 | 9.58E-21 |  |  |
|  | cg04571143 | 15 | 99396721 | 99396723 | *LINC02244;RP11-20G13.2* | -72.46 | 7.62 | 1.08E-20 |  |  |
|  | cg02644966 | 17 | 76248002 | 76248004 | *NA* | -105.80 | 11.22 | 2.39E-20 |  |  |
|  | cg05365685 | 3 | 10394721 | 10394723 | *ATP2B2;MIR885* | -77.74 | 8.41 | 1.18E-19 |  |  |
|  | cg13314036 | 14 | 58365142 | 58365144 | *ARID4A* | -78.28 | 8.51 | 1.79E-19 |  |  |
|  | cg07891369 | 2 | 9009829 | 9009831 | *NA* | -78.09 | 8.64 | 6.80E-19 |  |  |
|  | cg16132835 | 7 | 133275047 | 133275049 | *EXOC4* | -60.31 | 6.84 | 4.20E-18 |  |  |
|  | cg23699737 | 20 | 50131758 | 50131760 | *TMEM189;TMEM189-UBE2V1* | -58.33 | 6.67 | 8.19E-18 |  |  |
|  | cg16672408 | 18 | 55347727 | 55347729 | *TCF4* | -92.64 | 10.63 | 1.02E-17 |  |  |
|  | cg25748357 | 7 | 50732280 | 50732282 | *GRB10* | -57.40 | 6.75 | 5.80E-17 |  |  |
|  | cg01758046 | 1 | 21257084 | 21257086 | *ECE1* | -60.88 | 7.24 | 1.20E-16 |  |  |
|  | cg24868453 | 12 | 2904585 | 2904587 | *TULP3* | -66.89 | 7.96 | 1.35E-16 |  |  |
|  | cg11277456 | 1 | 109261073 | 109261075 | *CELSR2* | -94.33 | 11.32 | 2.35E-16 | S shore |  |
|  | cg20203534 | 6 | 1629823 | 1629825 | *GMDS* | -54.10 | 6.56 | 4.38E-16 |  |  |
|  | cg17069165 | 17 | 49814828 | 49814830 | *KAT7* | 62.70 | 7.65 | 6.76E-16 |  |  |
|  | cg20949846 | 12 | 10934413 | 10934415 | *PRH1;PRH1-PRR4;PRR4* | -53.84 | 6.68 | 1.91E-15 |  |  |
|  | cg09530173 | 14 | 96098320 | 96098322 | *NA* | 42.60 | 5.31 | 2.57E-15 |  |  |
|  | cg11548242 | 5 | 141764900 | 141764902 | *NA* | -74.17 | 9.39 | 6.63E-15 |  |  |
|  | cg07327587 | 16 | 50645695 | 50645697 | *RP11-401P9.4* | -64.41 | 8.16 | 6.86E-15 |  |  |
|  | cg16276452 | 14 | 23072676 | 23072678 | *ACIN1* | -52.38 | 6.72 | 1.49E-14 |  |  |
|  | cg17394331 | 12 | 68246506 | 68246508 | *NA* | -99.70 | 12.87 | 2.07E-14 |  |  |
|  | cg17297421 | 6 | 142132466 | 142132468 | *GJE1* | -50.12 | 6.61 | 7.29E-14 |  |  |
|  | cg01710244 | 5 | 65779776 | 65779778 | *NLN* | 60.53 | 7.99 | 7.35E-14 |  |  |
|  | cg09015231 | 21 | 27954470 | 27954472 | *AJ006995.3* | -44.02 | 5.95 | 2.75E-13 |  |  |
|  | cg18803843 | 2 | 85590832 | 85590834 | *VAMP5* | -111.21 | 15.09 | 3.28E-13 |  |  |
|  | cg08641767 | 10 | 72483669 | 72483671 | *MICU1;RP11-205A8.3* | -131.38 | 17.95 | 4.83E-13 |  |  |
|  | cg07914385 | 6 | 20727851 | 20727853 | *CDKAL1* | -50.11 | 6.89 | 6.41E-13 |  |  |
|  | cg26656977 | 6 | 100225140 | 100225142 | *NA* | -58.42 | 8.45 | 7.97E-12 | N shelf |  |
|  | cg20138617 | 10 | 96491880 | 96491882 | *TLL2* | -35.82 | 5.22 | 1.09E-11 |  |  |
|  | cg05006728 | 3 | 98731866 | 98731868 | *ST3GAL6;ST3GAL6-AS1* | -110.44 | 16.13 | 1.25E-11 | N shore |  |
|  | cg03723356 | 17 | 47970393 | 47970395 | *CDK5RAP3;RP11-6N17.9* | -105.78 | 15.48 | 1.35E-11 | N shore | Promoter |
|  | cg14559492 | 1 | 107571876 | 107571878 | *VAV3* | -65.84 | 9.64 | 1.37E-11 |  |  |
|  | cg27025276 | 12 | 65634320 | 65634322 | *RP11-230G5.2* | -51.02 | 7.55 | 2.29E-11 |  |  |
|  | cg13522114 | 19 | 6216393 | 6216395 | *MLLT1* | -102.88 | 15.53 | 5.37E-11 |  |  |
|  | cg11070916 | 14 | 100296964 | 100296966 | *SLC25A29* | -61.40 | 9.28 | 5.67E-11 | S shelf |  |
|  | cg00543759 | 5 | 3048447 | 3048449 | *NA* | -40.99 | 6.20 | 6.09E-11 |  |  |
|  | cg07187840 | 8 | 94217442 | 94217444 | *CDH17* | 80.48 | 12.18 | 6.13E-11 |  |  |
|  | cg21531176 | 19 | 16883287 | 16883289 | *NA* | -30.63 | 4.67 | 8.26E-11 |  |  |
|  | cg16323371 | 16 | 70268226 | 70268228 | *AARS* | -43.62 | 6.68 | 1.01E-10 |  |  |
|  | cg07529381 | 17 | 47970400 | 47970402 | *CDK5RAP3;RP11-6N17.9* | -125.88 | 19.75 | 2.71E-10 | N shore | Promoter |
|  | cg11674865 | 1 | 161621697 | 161621699 | *FCGR2B* | 45.11 | 7.12 | 3.45E-10 |  |  |
|  | cg27295561 | 17 | 75758426 | 75758428 | *GALK1* | -79.33 | 12.55 | 3.79E-10 |  |  |
|  | cg22165275 | 17 | 41226198 | 41226200 | *KRTAP9-2;KRTAP9-9* | 41.51 | 6.62 | 5.09E-10 |  |  |
|  | cg14373199 | 18 | 37890185 | 37890187 | *NA* | -46.68 | 7.45 | 5.41E-10 |  |  |
|  | cg06159872 | 1 | 161632953 | 161632955 | *FCGR2B;FCGR3B* | -69.07 | 11.07 | 6.13E-10 |  |  |
|  | cg27013765 | 3 | 197675135 | 197675137 | *MIR922;RUBCN* | 39.11 | 6.27 | 6.25E-10 |  |  |
|  | cg13062424 | 12 | 123253383 | 123253385 | *C12orf65;RP11-282O18.3* | -36.29 | 5.88 | 9.49E-10 |  |  |
|  | cg12587729 | 12 | 49741137 | 49741139 | *TMBIM6* | 71.09 | 11.54 | 1.02E-09 |  |  |
|  | cg02252404 | 16 | 89958315 | 89958317 | *DEF8* | -37.18 | 6.10 | 1.50E-09 |  |  |
|  | cg00957952 | 2 | 7898165 | 7898167 | *AC007463.2* | -39.77 | 6.54 | 1.66E-09 |  |  |
|  | cg16466932 | 11 | 69658521 | 69658523 | *LTO1* | -32.15 | 5.33 | 2.21E-09 |  |  |
|  | cg12829016 | 12 | 107649375 | 107649377 | *BTBD11;Y_RNA* | 62.88 | 10.49 | 2.80E-09 |  |  |
|  | cg09303161 | 2 | 191677871 | 191677873 | *NABP1* | -84.65 | 14.13 | 2.82E-09 |  |  |
|  | cg06919231 | 6 | 2432049 | 2432051 | *GMDS-DT* | -27.10 | 4.58 | 4.47E-09 |  |  |
|  | cg19884600 | 1 | 158932147 | 158932149 | *PYHIN1* | -44.38 | 7.60 | 6.86E-09 |  |  |
|  | cg07878951 | 16 | 48142488 | 48142490 | *ABCC12* | -37.84 | 6.48 | 6.89E-09 |  |  |
|  | cg06375652 | 16 | 86066816 | 86066818 | *NA* | -53.06 | 9.11 | 7.53E-09 |  |  |
|  | cg10505610 | 5 | 172355202 | 172355204 | *SH3PXD2B* | 41.00 | 7.08 | 9.25E-09 |  |  |
|  | cg18837178 | 5 | 12790916 | 12790918 | *LINC01194* | -28.87 | 5.08 | 1.71E-08 | N shelf |  |
|  | cg17014374 | 4 | 157290899 | 157290901 | *GRIA2* | -32.93 | 5.80 | 1.78E-08 |  |  |
|  | cg02106043 | 1 | 161605528 | 161605530 | *FCGR2B;FCGR2C;HSPA7* | -31.06 | 5.48 | 1.89E-08 | N shore |  |
|  | cg24517800 | 6 | 43021618 | 43021620 | *RRP36* | 56.71 | 10.02 | 1.93E-08 |  |  |
|  | cg09900172 | 2 | 145043886 | 145043888 | *TEX41* | -30.73 | 5.45 | 2.18E-08 |  |  |
|  | cg19375371 | 3 | 45979670 | 45979672 | *FYCO1* | 63.92 | 11.37 | 2.39E-08 |  |  |
|  | cg01550473 | 1 | 161524141 | 161524143 | *HSPA6;RP11-25K21.6* | -58.48 | 10.43 | 2.59E-08 |  |  |
|  | cg08458758 | 12 | 124882840 | 124882842 | *SCARB1* | -50.07 | 8.98 | 3.03E-08 |  |  |
|  | cg06336250 | 1 | 229894258 | 229894260 | *NA* | -84.98 | 15.40 | 4.24E-08 |  |  |
|  | cg10101263 | 11 | 130916737 | 130916739 | *SNX19* | 69.25 | 12.57 | 4.46E-08 |  |  |
|  | cg09492247 | 13 | 91722486 | 91722488 | *GPC5* | 56.53 | 10.27 | 4.55E-08 |  |  |
|  | cg21619867 | 17 | 4938584 | 4938586 | *RNF167;SLC25A11* | 35.25 | 6.41 | 4.64E-08 | N shore |  |
|  | cg25340919 | 2 | 230580865 | 230580867 | *AC010149.4* | -28.70 | 5.22 | 4.72E-08 |  |  |
|  | cg18403193 | 6 | 165936488 | 165936490 | *LINC00473;PDE10A* | -81.48 | 14.89 | 5.53E-08 | N shelf |  |

Abbreviations: EWAS, epigenome-wide association study; chr, chromosome; start, CpG start position in GRCh38/hg38; end, CpG end position in GRCh38/hg38; SE, standard error; HDL, high-density lipoprotein; LDL, low-density lipoprotein; TG, triglycerides; TC, total cholesterol.

Additional file 1: Table S4. Significant CpGs in outcome EWAS of blood lipid among survivors of African ancestry in SJLIFE cohort (*P*<9x10^-8^).

| **EWAS** | **CpG** | **chr** | **start** | **end** | **Nearby Gene** | **Effect size** | **SE** | ***P*** | **UCSC CpG Island** | **Regulatory Feature group** |
| --- | --- | --- | --- | --- | --- | --- | --- | --- | --- | --- |
| TG exposure | cg26675329 | 2 | 102417292 | 102417294 | *IL18RAP* | -3.35E-03 | 5.48E-04 | 3.12E-08 |  |  |
|  | cg06738121 | 2 | 239576290 | 239576292 | NA | -4.68E-03 | 6.73E-04 | 7.68E-10 |  |  |
|  | cg04747445 | 3 | 107522569 | 107522571 | *BBX* | 4.66E-03 | 7.17E-04 | 5.67E-09 | N Shore |  |
|  | cg05416955 | 9 | 136371273 | 136371275 | *CARD9* | -3.87E-03 | 5.83E-04 | 2.98E-09 | Island |  |
|  | cg21376908 | 17 | 57305062 | 57305064 | *MSI2* | -2.70E-03 | 4.58E-04 | 8.08E-08 |  |  |
|  | cg16197879 | 21 | 36467029 | 36467031 | *AP000695.4; AP000695.6; CLDN14* | -4.18E-03 | 6.81E-04 | 2.78E-08 |  |  |
| HDL outcome | cg14558275 | 7 | 106865406 | 106865408 | *PIK3CG* | -14.69 | 2.61 | 6.48E-08 | N Shelf | Promoter |
| LDL outcome | cg16411101 | 1 | 54365979 | 54365981 | *SSBP3* | 25.33 | 4.37 | 2.69E-08 |  |  |
| TC outcome | cg16411101 | 1 | 54365979 | 54365981 | *SSBP3* | 38.27 | 6.39 | 1.05E-08 |  |  |
|  | cg23724016 | 11 | 617707 | 617709 | *CDHR5* | 19.96 | 3.32 | 8.86E-09 | Island |  |
| TG outcome | cg05416345 | 1 | 18939692 | 18939694 | *IFFO2* | 41.85 | 7.43 | 6.31E-08 |  |  |
|  | cg04348872 | 2 | 24918826 | 24918828 | *ADCY3* | 44.86 | 7.84 | 3.97E-08 | N Shore |  |
|  | cg12571727 | 2 | 75960378 | 75960380 | NA | 36.35 | 6.35 | 4.02E-08 |  |  |
|  | cg01111718 | 11 | 105891647 | 105891649 | *GRIA4* | -24.58 | 4.33 | 4.99E-08 |  |  |
|  | cg12686539 | 12 | 133108175 | 133108177 | *ZNF891* | 25.32 | 4.35 | 2.39E-08 |  |  |

Abbreviations: EWAS, epigenome-wide association study; chr, chromosome; start, CpG start position in GRCh38/hg38; end, CpG end position in GRCh38/hg38; SE, standard error; TG, triglycerides; TC, total cholesterol; HDL, high-density lipoprotein; LDL, low-density lipoprotein.

Additional file 1: Table S5. SJLIFE meta-analysis of blood lipids EWAS among survivors of European and African ancestry using METAL (*P*<9x10^-8^).

| **EWAS** | **CpG** | **Chr_hg38** | **Start_hg38** | **End_hg38** | **Nearby Gene** | **Effect** | **SE** | **P** | **Direction** | **I^2^** | **P_het_** |
| --- | --- | --- | --- | --- | --- | --- | --- | --- | --- | --- | --- |
| HDL exposure | *cg27324117 ^a^ | 4 | 153793134 | 153793136 | *NA* | -0.0024 | 0.0004 | 8.71E-08 | -- | 0 | 0.4765 |
|  | *cg02443467 ^a^ | 10 | 47191407 | 47191409 | *NA* | -0.0021 | 0.0004 | 2.30E-08 | -- | 0 | 0.5916 |
|  | *cg07373304 ^a^ | 16 | 998982 | 998984 | *RP11-161M6.3* | -0.0023 | 0.0004 | 1.41E-08 | -- | 36 | 0.2115 |
|  | cg14543017 ^a^ | 20 | 45944371 | 45944373 | *PCIF1* | 0.0024 | 0.0004 | 4.89E-08 | ++ | 10 | 0.292 |
| TC exposure | cg00574958 ^c^ | 11 | 68840153 | 68840155 | *CPT1A* | -0.002 | 0.0003 | 3.33E-10 | -+ | 85.8 | 8.04E-03 |
| TG exposure | ch.1.1262625R ^a^ | 1 | 39747240 | 39747241 | *PPIE* | 0.0006 | 0.0001 | 3.40E-09 | ++ | 0 | 0.5125 |
|  | cg03725309 ^a^ | 1 | 109214962 | 109214964 | *SARS* | -0.0006 | 0.0001 | 3.55E-13 | -- | 0 | 0.6425 |
|  | cg17250680 ^b^ | 2 | 226981934 | 226981936 | *RHBDD1* | 0.0003 | 0.0001 | 3.02E-08 | +- | 56.9 | 0.1279 |
|  | cg02658330 ^a^ | 3 | 58667865 | 58667867 | *FAM3D* | -0.0004 | 0.0001 | 1.92E-09 | -- | 0 | 0.6208 |
|  | cg07507418 ^a^ | 6 | 42404545 | 42404547 | *TRERF1* | -0.0003 | 0 | 1.10E-08 | -- | 0 | 0.9014 |
|  | ch.6.98859407F ^d^ | 6 | 98304809 | 98304810 | *RP11-436D23.1* | 0.0007 | 0.0001 | 1.74E-13 | ++ | 71.1 | 0.06297 |
|  | cg03982376 ^a^ | 6 | 149485522 | 149485524 | *ZC3H12D* | -0.0005 | 0.0001 | 1.11E-09 | -- | 0 | 0.4864 |
|  | cg01507280 ^b^ | 7 | 44472076 | 44472078 | *NUDCD3* | -0.0005 | 0.0001 | 1.19E-08 | -+ | 0 | 0.3201 |
|  | cg12397924 ^c^ | 7 | 94657873 | 94657875 | *PEG10* | -0.0004 | 0.0001 | 5.61E-08 | -+ | 84.2 | 0.01185 |
|  | cg03360355 ^a^ | 9 | 135352663 | 135352665 | *RP11-555H7.2* | -0.0003 | 0 | 2.38E-09 | -- | 0 | 0.6882 |
|  | cg12726743 ^a^ | 10 | 73875089 | 73875091 | *CAMK2G* | -0.0004 | 0.0001 | 1.41E-08 | -- | 0 | 0.4735 |
|  | cg00574958 ^a^ | 11 | 68840153 | 68840155 | *CPT1A* | -0.0013 | 0.0001 | 3.50E-28 | -- | 45.7 | 0.1746 |
|  | cg09737197 ^a^ | 11 | 68840206 | 68840208 | *CPT1A* | -0.0007 | 0.0001 | 1.03E-10 | -- | 47.5 | 0.1677 |
|  | cg05325763 ^a^ | 11 | 68840250 | 68840252 | *CPT1A* | -0.0012 | 0.0001 | 2.38E-23 | -- | 35.9 | 0.2116 |
|  | cg17058475 ^a^ | 11 | 68840268 | 68840270 | *CPT1A* | -0.0011 | 0.0001 | 1.32E-18 | -- | 27.9 | 0.2388 |
|  | *cg01082498 ^a^ | 11 | 68840756 | 68840758 | *CPT1A* | -0.0006 | 0.0001 | 7.23E-08 | -- | 0 | 0.8322 |
|  | cg19120513 ^a^ | 11 | 102318571 | 102318573 | *BIRC3* | -0.0003 | 0.0001 | 1.09E-08 | -- | 0 | 0.4472 |
|  | cg01620154 ^b^ | 12 | 47798315 | 47798317 | *HDAC7* | 0.0003 | 0.0001 | 6.26E-08 | +- | 43.9 | 0.182 |
|  | cg10417863 ^a^ | 12 | 132780368 | 132780370 | *GOLGA3* | -0.0003 | 0.0001 | 1.30E-09 | -- | 0 | 0.5526 |
|  | cg05032656 ^a^ | 14 | 23729804 | 23729806 | *RP11-388E23.2* | -0.0005 | 0.0001 | 2.04E-08 | -- | 0 | 0.4984 |
|  | cg12604154 ^a^ | 15 | 45390179 | 45390181 | *GATM* | -0.0003 | 0.0001 | 1.85E-08 | -- | 0 | 0.7824 |
|  | cg24327132 ^b^ | 15 | 72228290 | 72228292 | *PKM* | -0.0004 | 0.0001 | 5.26E-09 | -+ | 60.7 | 0.1108 |
|  | cg25600227 ^a^ | 15 | 78193555 | 78193557 | *ACSBG1* | -0.0004 | 0.0001 | 2.59E-10 | -- | 0 | 0.8482 |
|  | cg10886794 ^a^ | 16 | 290026 | 290028 | *AXIN1* | -0.0004 | 0.0001 | 2.75E-09 | -- | 0 | 0.3979 |
|  | *cg06004232 ^a^ | 16 | 11293335 | 11293337 | *RMI2* | 0.0003 | 0.0001 | 1.79E-08 | ++ | 5.7 | 0.3032 |
|  | cg23623659 ^a^ | 17 | 5022179 | 5022181 | *KIF1C* | -0.0009 | 0.0002 | 6.07E-10 | -- | 0 | 0.9594 |
|  | cg18807499 ^b^ | 17 | 57096382 | 57096384 | *AKAP1;RP11-166P13.4* | -0.0003 | 0.0001 | 1.26E-08 | -+ | 62.4 | 0.1029 |
|  | cg07616376 ^b^ | 18 | 2980516 | 2980518 | *LPIN2;RP11-737O24.1* | -0.0006 | 0.0001 | 7.52E-08 | -+ | 37.5 | 0.2057 |
|  | cg10567810 ^a^ | 18 | 11149068 | 11149070 | *PIEZO2* | 0.0007 | 0.0001 | 7.21E-12 | ++ | 5.7 | 0.3031 |
|  | *cg11686214 ^a^ | 19 | 58573616 | 58573618 | *CENPBD1P1;MZF1;MZF1-AS1* | 0.0006 | 0.0001 | 5.20E-08 | ++ | 0 | 0.929 |
|  | cg09097291 ^b^ | 20 | 1170871 | 1170873 | *PSMF1* | -0.0005 | 0.0001 | 1.67E-08 | -+ | 0 | 0.4764 |
|  | cg09720768 ^b^ | 20 | 50645405 | 50645407 | *RIPOR3;RP4-530I15.6* | -0.0004 | 0.0001 | 4.31E-10 | -+ | 34.2 | 0.2175 |
|  | cg16740586 ^a^ | 21 | 42235808 | 42235810 | *ABCG1* | 0.0005 | 0.0001 | 2.01E-09 | ++ | 11.5 | 0.2879 |
| HDL outcome | ch.1.829344F ^c^ | 1 | 24657005 | 24657006 | *SRRM1* | 5.4291 | 0.8434 | 1.22E-10 | +- | 92 | 4.14E-04 |
|  | cg21750129 ^c^ | 9 | 71249778 | 71249780 | *TRPM3* | 5.9543 | 0.972 | 9.03E-10 | +- | 85.7 | 8.16E-03 |
| LDL outcome | ch.1.829344F ^c^ | 1 | 24657005 | 24657006 | *SRRM1* | 14.1591 | 2.0281 | 2.92E-12 | +- | 91.3 | 7.10E-04 |
| TC outcome | ch.1.829344F ^c^ | 1 | 24657005 | 24657006 | *SRRM1* | 20.7496 | 2.7463 | 4.18E-14 | +- | 95.2 | 5.10E-06 |
|  | cg21750129 ^c^ | 9 | 71249778 | 71249780 | *TRPM3* | 17.5186 | 3.1528 | 2.75E-08 | +- | 95 | 8.25E-06 |
| TG outcome | cg01758046 ^c^ | 1 | 21257084 | 21257086 | *ECE1* | -29.5083 | 5.3277 | 3.05E-08 | -+ | 97.6 | 1.48E-10 |
|  | cg11505417 ^c^ | 1 | 22639557 | 22639559 | *C1QA* | 23.3109 | 3.4561 | 1.53E-11 | +- | 98.9 | 6.98E-21 |
|  | cg13695997 ^d^ | 1 | 81525284 | 81525286 | *ADGRL2;RP5-837I24.2* | -65.3892 | 7.3505 | 5.80E-19 | -- | 98.6 | 9.72E-17 |
|  | cg07891369 ^d^ | 2 | 9009829 | 9009831 | *NA* | -37.6042 | 5.0878 | 1.46E-13 | -- | 97 | 6.67E-09 |
|  | *cg23550429 ^a^ | 2 | 30797935 | 30797937 | *CAPN13* | -15.4833 | 2.8087 | 3.54E-08 | -- | 34.9 | 0.2152 |
|  | cg00656366 ^c^ | 2 | 128550034 | 128550036 | *NA* | -48.3437 | 7.9972 | 1.49E-09 | -+ | 99.2 | 2.61E-29 |
|  | *cg03260858 ^a^ | 3 | 49103337 | 49103339 | *QARS* | 34.7521 | 6.1816 | 1.89E-08 | ++ | 31.4 | 0.2272 |
|  | cg01233571 ^c^ | 5 | 60651660 | 60651662 | *DEPDC1B* | -31.031 | 5.1646 | 1.87E-09 | -+ | 98.9 | 4.06E-21 |
|  | cg11548242 ^c^ | 5 | 141764900 | 141764902 | *NA* | -37.6461 | 6.9979 | 7.46E-08 | -+ | 97.1 | 5.35E-09 |
|  | cg20203534 ^d^ | 6 | 1629823 | 1629825 | *GMDS* | -24.0714 | 4.1599 | 7.19E-09 | -- | 97.2 | 3.12E-09 |
|  | cg16132835 ^d^ | 7 | 133275047 | 133275049 | *EXOC4* | -27.0988 | 4.5239 | 2.10E-09 | -- | 97.6 | 9.06E-11 |
|  | cg17960556 ^c^ | 7 | 157044322 | 157044324 | *NA* | -40.111 | 6.7842 | 3.37E-09 | -+ | 98.5 | 5.87E-16 |
|  | cg15295857 ^c^ | 8 | 144448140 | 144448142 | *NA* | -52.7459 | 8.9284 | 3.47E-09 | -+ | 99.6 | 3.02E-55 |
|  | cg05453148 ^d^ | 9 | 87607479 | 87607481 | *DAPK1* | -23.3996 | 4.15 | 1.72E-08 | -- | 99.3 | 7.69E-34 |
|  | cg26727817 ^c^ | 10 | 121617326 | 121617328 | *RP11-62L18.3* | -30.2249 | 5.5836 | 6.19E-08 | -+ | 98.4 | 3.12E-15 |
|  | *cg20601481 ^a^ | 11 | 67288314 | 67288316 | *ANKRD13D* | -34.4364 | 6.2857 | 4.29E-08 | -- | 0 | 0.4668 |
|  | cg08657939 ^c^ | 11 | 69480238 | 69480240 | *AP000439.3* | -39.8705 | 7.4344 | 8.19E-08 | -+ | 99.5 | 2.22E-47 |
|  | cg01111718 ^a^ | 11 | 105891647 | 105891649 | *GRIA4* | -21.8982 | 3.9803 | 3.76E-08 | -- | 59.8 | 0.1146 |
|  | cg24868453 ^c^ | 12 | 2904585 | 2904587 | *TULP3* | -33.2445 | 6.1152 | 5.44E-08 | -+ | 97.7 | 4.22E-11 |
|  | cg11570494 ^c^ | 12 | 42235171 | 42235173 | *YAF2* | -43.7383 | 6.2526 | 2.65E-12 | -+ | 99.2 | 2.65E-28 |
|  | cg20054394 ^c^ | 12 | 132361751 | 132361753 | *RP13-895J2.11* | -40.339 | 6.9129 | 5.37E-09 | -+ | 98.8 | 7.20E-20 |
|  | *cg19590858 ^a^ | 14 | 34540645 | 34540647 | *EAPP;RP11-671J11.5* | -28.9022 | 5.3768 | 7.64E-08 | -- | 0 | 0.6798 |
|  | cg20703271 ^c^ | 15 | 41822236 | 41822238 | *MAPKBP1* | -52.8235 | 7.6062 | 3.79E-12 | -+ | 99.3 | 1.11E-31 |
|  | cg04571143 ^c^ | 15 | 99396721 | 99396723 | *LINC02244;RP11-20G13.2* | -28.1041 | 5.1127 | 3.87E-08 | -+ | 98.4 | 3.85E-15 |
|  | cg17069165 ^d^ | 17 | 49814828 | 49814830 | *KAT7* | 28.3034 | 4.7192 | 2.00E-09 | ++ | 96.9 | 1.11E-08 |
|  | *cg20433103 ^a^ | 17 | 63941062 | 63941064 | *SCN4A* | 38.6549 | 6.8825 | 1.95E-08 | ++ | 0 | 0.6438 |
|  | *cg07439166 ^a^ | 19 | 35902502 | 35902504 | *AD000864.6;HCST;NFKBID* | -37.3876 | 6.666 | 2.04E-08 | -- | 6.6 | 0.3009 |
|  | cg26471866 ^c^ | 19 | 55542146 | 55542148 | *SBK3* | -61.5558 | 8.4447 | 3.12E-13 | -+ | 98.8 | 1.94E-20 |
|  | cg23699737 ^c^ | 20 | 50131758 | 50131760 | *TMEM189;TMEM189-UBE2V1* | -29.4224 | 5.1533 | 1.13E-08 | -+ | 97.9 | 8.91E-12 |
|  | cg18452324 ^c^ | 22 | 37486358 | 37486360 | *MFNG* | 42.9594 | 4.0552 | 3.19E-26 | +- | 99.7 | 3.87E-72 |
|  | cg10769317 ^d^ | 22 | 42344268 | 42344270 | *TCF20* | 22.6776 | 3.1562 | 6.72E-13 | ++ | 99.1 | 1.93E-26 |

*Additional significant associations in meta-EWAS comparing to EWAS results among survivors of European ancestry or African ancestry.

^a^ CpGs with P_het_>0.1 and the same direction between survivors of European and African ancestry;

^b^ CpGs with P_het_>0.1 and different directions between survivors of European and African ancestry;

^c^ CpGs with P_het_<0.1 and different directions between survivors of European and African ancestry;

^d^ CpGs with P_het_<0.1 and the same direction between survivors of European and African ancestry.

Additional file 1: Table S6. Previous EWAS results (reported in EWAS Catalog with *P*<9x10^-8^ and tissue including blood) for 148 blood lipids associated CpG hits (among EA and AA) in the current study.

| Survivors | CpG | Chr | Pos | Nearby Gene | Beta | SE | P | Trait | PMID | Tissue |
| --- | --- | --- | --- | --- | --- | --- | --- | --- | --- | --- |
| EA | cg11505417 | 1 | 22966051 | *C1QA* | -8.59E-02 | 2.59E-03 | 1.00E-81 | Tissue | 30602389 | Buccal cells and peripheral blood mononuclear cells |
| EA | ch.1.829344F | 1 | 24983497 | *SRRM1* | 1.97E-02 | 3.20E-03 | 5.84E-10 | Rheumatoid arthritis | 23334450 | Whole blood |
| EA | ch.1.1262625R | 1 | 40212913 | *PPIE* | NA | NA | 6.39E-08 | Primary Sjogrens syndrome | 26857698 | Whole blood |
| EA | cg03725309 | 1 | 109757585 | *SARS* | -7.80E-04 | 8.80E-05 | 5.20E-18 | Age | 23177740 | Whole blood |
| EA | cg03725309 | 1 | 109757585 | *SARS* | 5.50E+00 | 1.22E-01 | 4.40E-11 | M32197_3-4-hydroxyphenyllactate | 24014485 | Whole blood |
| EA | cg03725309 | 1 | 109757585 | *SARS* | 5.60E+00 | 1.23E-01 | 3.70E-11 | M32197_3-4-hydroxyphenyllactate | 24014485 | Whole blood |
| EA | cg03725309 | 1 | 109757585 | *SARS* | -9.76E-01 | 7.25E-02 | 3.00E-41 | Ageing | 25888029 | Whole blood |
| EA | cg03725309 | 1 | 109757585 | *SARS* | NA | NA | 2.86E-12 | Primary Sjogrens syndrome | 26857698 | Whole blood |
| EA | cg03725309 | 1 | 109757585 | *SARS* | -1.03E-03 | 1.57E-04 | 7.37E-11 | Gestational age | 27717397 | Cord blood |
| EA | cg03725309 | 1 | 109757585 | *SARS* | -2.20E-03 | 3.53E-04 | 4.54E-10 | C-reactive protein | 27955697 | Whole blood |
| EA | cg03725309 | 1 | 109757585 | *SARS* | -2.48E+01 | 3.20E+00 | 3.80E-13 | Body mass index | 28002404 | Whole blood |
| EA | cg03725309 | 1 | 109757585 | *SARS* | NA | NA | 9.00E-19 | Body mass index | 28002404 | Whole blood |
| EA | cg03725309 | 1 | 109757585 | *SARS* | -2.30E+01 | 3.08E+00 | 9.50E-14 | Body mass index | 28002404 | Whole blood |
| EA | cg03725309 | 1 | 109757585 | *SARS* | NA | NA | 1.44E-49 | Age 4 vs age 0 | 28056824 | Whole blood, cord blood |
| EA | cg03725309 | 1 | 109757585 | *SARS* | NA | NA | 3.93E-09 | Body mass index | 28095459 | Whole blood |
| EA | cg03725309 | 1 | 109757585 | *SARS* | -9.05E-03 | 1.21E-03 | 7.11E-14 | Serum triglycerides | 28213390 | Whole blood |
| EA | cg03725309 | 1 | 109757585 | *SARS* | -7.84E-03 | 1.26E-03 | 4.56E-10 | Serum triglycerides | 28213390 | Whole blood |
| EA | cg03725309 | 1 | 109757585 | *SARS* | -9.71E-03 | 9.95E-04 | 1.56E-22 | Serum triglycerides | 28213390 | Whole blood |
| EA | cg03725309 | 1 | 109757585 | *SARS* | -8.40E-03 | 1.00E-03 | 4.31E-16 | Serum triglycerides | 28213390 | Whole blood |
| EA | cg03725309 | 1 | 109757585 | *SARS* | -1.11E-02 | 1.75E-03 | 2.09E-10 | Serum triglycerides | 28213390 | Whole blood |
| EA | cg03725309 | 1 | 109757585 | *SARS* | -1.17E-02 | 2.09E-03 | 1.02E-08 | Age | 28811542 | Whole blood |
| EA | cg03725309 | 1 | 109757585 | *SARS* | -1.00E-04 | NA | 3.20E-17 | Systolic Blood Pressure | 29198723 | Whole blood, CD4+ T cells |
| EA | cg03725309 | 1 | 109757585 | *SARS* | -2.68E-01 | NA | 3.40E-12 | BMI | 29278407 | Whole blood |
| EA | cg03725309 | 1 | 109757585 | *SARS* | -1.89E-02 | NA | 2.50E-09 | Aging | 30264654 | Whole blood |
| EA | cg03725309 | 1 | 109757585 | *SARS* | 4.01E-01 | 3.71E-03 | 1.50E-174 | Tissue | 30602389 | Buccal cells and peripheral blood mononuclear cells |
| EA | cg03725309 | 1 | 109757585 | *SARS* | NA | NA | 6.20E-10 | Hepatic Fat | 30936141 | Whole blood |
| EA | cg03725309 | 1 | 109757585 | *SARS* | NA | NA | 4.60E-10 | Hepatic Fat | 30936141 | Whole blood |
| EA | cg03725309 | 1 | 109757585 | *SARS* | -1.90E-02 | 3.40E-03 | 5.00E-08 | Age | 31426852 | Whole Blood |
| EA | cg03725309 | 1 | 109757585 | *SARS* | -4.05E-03 | 7.40E-04 | 4.50E-08 | Smoking | 31536415 | Whole blood, CD4+ T cells, CD14+ monocytes |
| EA | cg03725309 | 1 | 109757585 | *SARS* | NA | NA | 2.60E-12 | Alcohol consumption | 31789449 | Whole blood |
| EA | cg03725309 | 1 | 109757585 | *SARS* | NA | NA | 4.10E-12 | Alcohol consumption | 31789449 | Whole blood |
| EA | cg03725309 | 1 | 109757585 | *SARS* | NA | NA | 1.70E-17 | Alcohol consumption | 31789449 | Whole blood |
| EA | cg03725309 | 1 | 109757585 | *SARS* | NA | NA | 6.20E-17 | Alcohol consumption | 31789449 | Whole blood |
| EA | cg03725309 | 1 | 109757585 | *SARS* | -3.50E-03 | 2.00E-04 | 0 | age | 33450751 | Whole blood |
| EA | cg19884600 | 1 | 158901938 | *PYHIN1* | -9.48E-01 | 1.25E-01 | 3.81E-14 | Ageing | 25888029 | Whole blood |
| EA | cg19884600 | 1 | 158901938 | *PYHIN1* | -4.53E-02 | 3.33E-03 | 5.10E-30 | Tissue | 30602389 | Buccal cells and peripheral blood mononuclear cells |
| EA | cg01550473 | 1 | 161493932 | *HSPA6* | 9.67E-02 | 3.29E-03 | 4.00E-73 | Tissue | 30602389 | Buccal cells and peripheral blood mononuclear cells |
| EA | cg01550473 | 1 | 161493932 | *HSPA6* | -8.90E-04 | 1.30E-04 | 1.60E-11 | age | 33450751 | Whole blood |
| EA | cg01550473 | 1 | 161493932 | *HSPA6* | NA | NA | 9.00E-09 | Gene expression of Affymetrix ID: 2440943 (gene symbol: FCGR3B) | 33752734 | Whole blood |
| EA | cg17405122 | 1 | 161494852 | *HSPA6* | -1.20E-03 | 1.40E-04 | 8.90E-16 | age | 33450751 | Whole blood |
| EA | cg02106043 | 1 | 161575319 | *HSPA7* | -1.40E-03 | 1.40E-04 | 0 | age | 33450751 | Whole blood |
| EA | cg02106043 | 1 | 161575319 | *HSPA7* | 1.50E-03 | NA | 6.90E-23 | age | 33450751 | Whole blood |
| EA | cg02106043 | 1 | 161575319 | *HSPA7* | 3.90E-03 | NA | 1.50E-09 | age | 33450751 | Whole blood |
| EA | cg13495918 | 1 | 161575324 | *HSPA7* | -1.97E-02 | 2.80E-03 | 2.10E-12 | Rheumatoid arthritis | 23334450 | Whole blood |
| EA | cg13495918 | 1 | 161575324 | *HSPA7* | -1.40E-03 | 1.60E-04 | 0 | age | 33450751 | Whole blood |
| EA | cg13495918 | 1 | 161575324 | *HSPA7* | 1.70E-03 | NA | 8.00E-19 | age | 33450751 | Whole blood |
| EA | cg11674865 | 1 | 161591488 | *-* | 3.80E-04 | NA | 4.10E-21 | age | 33450751 | Whole blood |
| EA | cg07891369 | 2 | 9149959 | *-* | -2.20E-02 | 2.77E-03 | 1.50E-13 | Tissue | 30602389 | Buccal cells and peripheral blood mononuclear cells |
| EA | cg07891369 | 2 | 9149959 | *-* | -1.30E-03 | 1.60E-04 | 1.80E-15 | age | 33450751 | Whole blood |
| EA | cg07891369 | 2 | 9149959 | *-* | 1.40E-03 | NA | 8.30E-23 | age | 33450751 | Whole blood |
| EA | cg09303161 | 2 | 192542598 | *OBFC2A* | 3.80E-04 | 6.00E-05 | 5.30E-10 | age | 33450751 | Whole blood |
| EA | cg05365685 | 3 | 10436406 | *ATP2B2;MIR885* | 1.80E-01 | 4.46E-03 | 4.20E-96 | Tissue | 30602389 | Buccal cells and peripheral blood mononuclear cells |
| EA | cg05365685 | 3 | 10436406 | *ATP2B2;MIR885* | -7.70E-04 | 1.30E-04 | 5.60E-09 | age | 33450751 | Whole blood |
| EA | cg02658330 | 3 | 58653593 | *FAM3D* | 1.40E-02 | 2.16E-03 | 8.40E-10 | Tissue | 30602389 | Buccal cells and peripheral blood mononuclear cells |
| EA | cg18837178 | 5 | 12791029 | *-* | 1.20E-03 | NA | 7.90E-09 | age | 33450751 | Whole blood |
| EA | cg10505610 | 5 | 171782207 | *SH3PXD2B* | -7.90E-04 | 7.60E-05 | 0 | age | 33450751 | Whole blood |
| EA | cg10505610 | 5 | 171782207 | *SH3PXD2B* | 6.70E-04 | NA | 5.20E-24 | age | 33450751 | Whole blood |
| EA | cg10505610 | 5 | 171782207 | *SH3PXD2B* | 2.20E-03 | NA | 1.60E-15 | age | 33450751 | Whole blood |
| EA | cg20203534 | 6 | 1630059 | *GMDS* | -2.60E-03 | 2.70E-04 | 0 | age | 33450751 | Whole blood |
| EA | cg20203534 | 6 | 1630059 | *GMDS* | 2.30E-03 | NA | 7.40E-17 | age | 33450751 | Whole blood |
| EA | cg07507418 | 6 | 42372284 | *TRERF1* | -1.94E-04 | 3.60E-05 | 7.15E-08 | Alcohol consumption per day | 27843151 | Whole blood |
| EA | cg07507418 | 6 | 42372284 | *TRERF1* | 1.42E-01 | 7.85E-03 | 1.90E-43 | Tissue | 30602389 | Buccal cells and peripheral blood mononuclear cells |
| EA | cg24517800 | 6 | 42989357 | *C6orf153* | 2.30E-03 | NA | 0 | age | 33450751 | Whole blood |
| EA | ch.6.98859407F | 6 | 98752686 | *-* | 6.70E-04 | NA | 2.80E-11 | age | 33450751 | Whole blood |
| EA | cg26656977 | 6 | 100673017 | *-* | -1.37E+00 | 2.56E-01 | 7.95E-08 | HIV infection | 27105112 | Whole blood |
| EA | cg26656977 | 6 | 100673017 | *-* | 3.72E-02 | 4.82E-03 | 6.00E-13 | Tissue | 30602389 | Buccal cells and peripheral blood mononuclear cells |
| EA | cg26656977 | 6 | 100673017 | *-* | -1.70E-03 | 2.10E-04 | 4.40E-16 | age | 33450751 | Whole blood |
| EA | cg18403193 | 6 | 166349977 | *C6orf176* | NA | NA | 1.22E-36 | Age 4 vs age 0 | 28056824 | Whole blood, cord blood |
| EA | cg18403193 | 6 | 166349977 | *C6orf176* | -5.97E-02 | 5.45E-03 | 4.80E-22 | Tissue | 30602389 | Buccal cells and peripheral blood mononuclear cells |
| EA | cg18403193 | 6 | 166349977 | *C6orf176* | -3.60E-03 | 1.80E-04 | 0 | age | 33450751 | Whole blood |
| EA | cg18403193 | 6 | 166349977 | *C6orf176* | 7.50E-03 | 1.20E-03 | 1.50E-09 | age | 33450751 | Whole blood |
| EA | cg18403193 | 6 | 166349977 | *C6orf176* | 9.60E-01 | 1.51E-01 | 1.50E-10 | schizophrenia | 33646943 | Whole blood |
| EA | cg18403193 | 6 | 166349977 | *C6orf176* | 9.70E-01 | 1.65E-01 | 5.00E-09 | schizophrenia | 33646943 | Whole blood |
| EA | cg20935223 | 7 | 6208238 | *CYTH3* | -5.70E-04 | 7.20E-05 | 2.20E-15 | age | 33450751 | Whole blood |
| EA | cg12397924 | 7 | 94287186 | *PEG10* | 1.34E+00 | 2.46E-01 | 5.43E-08 | HIV infection | 27105112 | Whole blood |
| EA | cg12397924 | 7 | 94287186 | *PEG10* | 2.23E-02 | 3.25E-03 | 8.60E-11 | Tissue | 30602389 | Buccal cells and peripheral blood mononuclear cells |
| EA | cg12397924 | 7 | 94287186 | *PEG10* | -1.70E-03 | 1.90E-04 | 0 | age | 33450751 | Whole blood |
| EA | cg17960556 | 7 | 156837017 | *-* | 4.83E-02 | 3.32E-03 | 8.20E-33 | Tissue | 30602389 | Buccal cells and peripheral blood mononuclear cells |
| EA | cg24040155 | 11 | 68200735 | *LRP5* | 1.23E-01 | 2.69E-03 | 2.60E-105 | Tissue | 30602389 | Buccal cells and peripheral blood mononuclear cells |
| EA | cg00574958 | 11 | 68607622 | *CPT1A* | -1.00E+00 | 5.10E-01 | 6.80E-14 | M33871_1-eicosadienoylglycerophosphocholine* | 24014485 | Whole blood |
| EA | cg00574958 | 11 | 68607622 | *CPT1A* | -1.00E+00 | 5.10E-01 | 9.20E-14 | M33871_1-eicosadienoylglycerophosphocholine* | 24014485 | Whole blood |
| EA | cg00574958 | 11 | 68607622 | *CPT1A* | -1.18E-01 | 1.60E-02 | 3.15E-13 | Triglycerides | 25583993 | Whole blood |
| EA | cg00574958 | 11 | 68607622 | *CPT1A* | -2.40E-03 | 4.00E-04 | 4.30E-08 | Body mass index change | 25935004 | Whole blood |
| EA | cg00574958 | 11 | 68607622 | *CPT1A* | -2.90E-03 | 4.00E-04 | 3.23E-12 | Body mass index | 25935004 | Whole blood |
| EA | cg00574958 | 11 | 68607622 | *CPT1A* | NA | NA | 3.44E-47 | Body mass index | 25935004 | Whole blood, CD4+ T cells |
| EA | cg00574958 | 11 | 68607622 | *CPT1A* | NA | NA | 5.77E-38 | Body mass index | 25935004 | Whole blood, CD4+ T cells |
| EA | cg00574958 | 11 | 68607622 | *CPT1A* | -3.40E-03 | 4.00E-04 | 5.79E-17 | Waist circumference | 25935004 | Whole blood |
| EA | cg00574958 | 11 | 68607622 | *CPT1A* | -4.00E-02 | 4.00E-03 | 2.40E-24 | Body mass index | 26110892 | Whole blood |
| EA | cg00574958 | 11 | 68607622 | *CPT1A* | NA | NA | 3.50E-37 | Body mass index | 26110892 | CD4+ T-cells, whole blood |
| EA | cg00574958 | 11 | 68607622 | *CPT1A* | -4.00E-03 | 7.00E-04 | 2.10E-08 | Waist circumference | 26110892 | Whole blood |
| EA | cg00574958 | 11 | 68607622 | *CPT1A* | NA | NA | 2.20E-16 | Waist circumference | 26110892 | CD4+ T-cells, Whole blood |
| EA | cg00574958 | 11 | 68607622 | *CPT1A* | 4.83E-01 | 6.06E-02 | 1.73E-15 | Hypertriglyceridemic waist | 26798409 | Whole blood |
| EA | cg00574958 | 11 | 68607622 | *CPT1A* | -2.73E-04 | 4.60E-05 | 4.20E-09 | Gestational age | 27717397 | Cord blood |
| EA | cg00574958 | 11 | 68607622 | *CPT1A* | -1.01E-04 | 1.50E-05 | 2.09E-11 | Alcohol consumption per day | 27843151 | Whole blood |
| EA | cg00574958 | 11 | 68607622 | *CPT1A* | -4.02E+01 | 3.10E+00 | 3.30E-33 | Body mass index | 28002404 | Whole blood |
| EA | cg00574958 | 11 | 68607622 | *CPT1A* | -2.13E+01 | 2.40E+00 | 2.80E-18 | Body mass index | 28002404 | Whole blood |
| EA | cg00574958 | 11 | 68607622 | *CPT1A* | NA | NA | 6.80E-49 | Body mass index | 28002404 | Whole blood |
| EA | cg00574958 | 11 | 68607622 | *CPT1A* | -4.21E+01 | 3.90E+00 | 3.50E-27 | Body mass index | 28002404 | Whole blood |
| EA | cg00574958 | 11 | 68607622 | *CPT1A* | -1.84E+01 | 2.52E+00 | 2.70E-13 | Body mass index | 28002404 | Whole blood |
| EA | cg00574958 | 11 | 68607622 | *CPT1A* | -3.78E+01 | 4.07E+00 | 4.90E-16 | Body mass index | 28002404 | Whole blood |
| EA | cg00574958 | 11 | 68607622 | *CPT1A* | -4.22E-02 | 4.03E-03 | 1.18E-24 | Body mass index | 28095459 | Whole blood |
| EA | cg00574958 | 11 | 68607622 | *CPT1A* | NA | NA | 2.20E-29 | Body mass index | 28095459 | Whole blood |
| EA | cg00574958 | 11 | 68607622 | *CPT1A* | -5.50E-02 | 4.00E-03 | 0 | Serum triglycerides | 28173150 | Whole blood |
| EA | cg00574958 | 11 | 68607622 | *CPT1A* | -7.50E-02 | 5.00E-03 | 0 | Serum triglycerides | 28173150 | Whole blood |
| EA | cg00574958 | 11 | 68607622 | *CPT1A* | -6.30E-02 | 4.00E-03 | 1.00E-44 | Serum triglycerides | 28173150 | Whole blood |
| EA | cg00574958 | 11 | 68607622 | *CPT1A* | -8.00E-02 | 5.00E-03 | 4.20E-56 | Serum triglycerides | 28173150 | Whole blood |
| EA | cg00574958 | 11 | 68607622 | *CPT1A* | -2.06E-02 | 2.60E-03 | 1.23E-15 | Triglycerides | 28194238 | Whole blood |
| EA | cg00574958 | 11 | 68607622 | *CPT1A* | -1.39E-02 | 1.30E-03 | 5.07E-26 | Triglycerides | 28194238 | Whole blood |
| EA | cg00574958 | 11 | 68607622 | *CPT1A* | -1.42E-02 | 1.30E-03 | 2.60E-26 | Triglycerides | 28194238 | Whole blood |
| EA | cg00574958 | 11 | 68607622 | *CPT1A* | -1.61E-02 | 1.60E-03 | 1.50E-22 | Triglycerides | 28194238 | Whole blood |
| EA | cg00574958 | 11 | 68607622 | *CPT1A* | -1.14E-02 | 1.40E-03 | 1.90E-16 | Triglycerides | 28194238 | Whole blood |
| EA | cg00574958 | 11 | 68607622 | *CPT1A* | -1.14E-02 | 1.60E-03 | 7.24E-13 | Triglycerides | 28194238 | Whole blood |
| EA | cg00574958 | 11 | 68607622 | *CPT1A* | -9.00E-03 | 7.24E-04 | 1.65E-35 | Serum triglycerides | 28213390 | Whole blood |
| EA | cg00574958 | 11 | 68607622 | *CPT1A* | -7.90E-03 | 7.50E-04 | 5.81E-26 | Serum triglycerides | 28213390 | Whole blood |
| EA | cg00574958 | 11 | 68607622 | *CPT1A* | -9.86E-03 | 6.07E-04 | 1.87E-59 | Serum triglycerides | 28213390 | Whole blood |
| EA | cg00574958 | 11 | 68607622 | *CPT1A* | -8.60E-03 | 6.00E-04 | 2.42E-42 | Serum triglycerides | 28213390 | Whole blood |
| EA | cg00574958 | 11 | 68607622 | *CPT1A* | -1.20E-02 | 1.13E-03 | 2.01E-26 | Serum triglycerides | 28213390 | Whole blood |
| EA | cg00574958 | 11 | 68607622 | *CPT1A* | -1.04E-02 | 1.18E-03 | 9.59E-19 | Serum triglycerides | 28213390 | Whole blood |
| EA | cg00574958 | 11 | 68607622 | *CPT1A* | -6.00E-03 | 1.00E-03 | 1.60E-08 | Gamma-glutamyl transferase | 28624579 | Whole blood |
| EA | cg00574958 | 11 | 68607622 | *CPT1A* | -8.70E-01 | 9.00E-02 | 4.20E-22 | Body mass index | 29099282 | Whole blood |
| EA | cg00574958 | 11 | 68607622 | *CPT1A* | -1.30E+00 | 1.10E-01 | 3.10E-32 | Body mass index | 29099282 | Whole blood |
| EA | cg00574958 | 11 | 68607622 | *CPT1A* | -2.50E+00 | 2.00E-01 | 7.50E-36 | Waist circumference | 29099282 | Whole blood |
| EA | cg00574958 | 11 | 68607622 | *CPT1A* | -3.90E+00 | 3.00E-01 | 1.20E-38 | Waist circumference | 29099282 | Whole blood |
| EA | cg00574958 | 11 | 68607622 | *CPT1A* | -7.50E-05 | NA | 3.00E-10 | Diastolic Blood Pressure | 29198723 | Whole blood, CD4+ T cells |
| EA | cg00574958 | 11 | 68607622 | *CPT1A* | -5.00E-05 | NA | 1.20E-13 | Systolic Blood Pressure | 29198723 | Whole blood, CD4+ T cells |
| EA | cg00574958 | 11 | 68607622 | *CPT1A* | -4.96E-01 | NA | 7.00E-21 | BMI | 29278407 | Whole blood |
| EA | cg00574958 | 11 | 68607622 | *CPT1A* | -6.41E-02 | 3.28E-03 | 1.60E-47 | Tissue | 30602389 | Buccal cells and peripheral blood mononuclear cells |
| EA | cg00574958 | 11 | 68607622 | *CPT1A* | NA | NA | 3.00E-11 | Hepatic Fat | 30936141 | Whole blood |
| EA | cg00574958 | 11 | 68607622 | *CPT1A* | NA | NA | 6.30E-11 | Hepatic Fat | 30936141 | Whole blood |
| EA | cg00574958 | 11 | 68607622 | *CPT1A* | -2.67E+00 | 3.50E-01 | 2.50E-14 | fasting glucose | 31197173 | whole blood |
| EA | cg00574958 | 11 | 68607622 | *CPT1A* | -1.89E+00 | 3.40E-01 | 2.20E-08 | fasting glucose | 31197173 | whole blood |
| EA | cg00574958 | 11 | 68607622 | *CPT1A* | -3.12E+00 | 3.90E-01 | 1.30E-15 | fasting insulin | 31197173 | whole blood |
| EA | cg00574958 | 11 | 68607622 | *CPT1A* | -3.70E-01 | NA | 5.20E-09 | Type II diabetes | 31506343 | Whole blood |
| EA | cg00574958 | 11 | 68607622 | *CPT1A* | -6.70E-03 | 7.90E-04 | 4.80E-17 | Type II diabetes | 31506343 | Whole blood |
| EA | cg00574958 | 11 | 68607622 | *CPT1A* | -9.20E-04 | NA | 3.00E-21 | body mass index | 31510868 | Whole blood |
| EA | cg00574958 | 11 | 68607622 | *CPT1A* | -9.20E-04 | NA | 5.90E-09 | body mass index | 31510868 | Whole blood |
| EA | cg00574958 | 11 | 68607622 | *CPT1A* | NA | NA | 4.00E-31 | body mass index | 31510868 | Whole blood |
| EA | cg00574958 | 11 | 68607622 | *CPT1A* | NA | NA | 8.20E-08 | Type II diabetes mellitus | 31691802 | Whole Blood |
| EA | cg00574958 | 11 | 68607622 | *CPT1A* | NA | NA | 9.60E-14 | Alcohol consumption | 31789449 | Whole blood |
| EA | cg00574958 | 11 | 68607622 | *CPT1A* | NA | NA | 5.40E-12 | Alcohol consumption | 31789449 | Whole blood |
| EA | cg00574958 | 11 | 68607622 | *CPT1A* | NA | NA | 8.30E-17 | Alcohol consumption | 31789449 | Whole blood |
| EA | cg00574958 | 11 | 68607622 | *CPT1A* | -2.67E-01 | 4.00E-02 | 2.30E-11 | waist circumference-to-height | 32901515 | Whole blood |
| EA | cg00574958 | 11 | 68607622 | *CPT1A* | -4.40E-01 | 6.10E-02 | 5.80E-13 | waist circumference-to-hip ratio | 32901515 | Whole blood |
| EA | cg00574958 | 11 | 68607622 | *CPT1A* | 5.80E-01 | 9.80E-02 | 4.30E-09 | L-LDL-FC_pct | 33413638 | Whole blood |
| EA | cg00574958 | 11 | 68607622 | *CPT1A* | -1.32E+01 | 2.34E+00 | 1.90E-08 | L-VLDL-TG | 33413638 | Whole blood |
| EA | cg00574958 | 11 | 68607622 | *CPT1A* | 1.20E+00 | 1.80E-01 | 9.10E-11 | M-VLDL-C_pct | 33413638 | Whole blood |
| EA | cg00574958 | 11 | 68607622 | *CPT1A* | 3.60E-01 | 4.60E-02 | 3.20E-15 | M-VLDL-PL_pct | 33413638 | Whole blood |
| EA | cg00574958 | 11 | 68607622 | *CPT1A* | -4.10E+00 | 3.50E-01 | 4.30E-33 | S-LDL-TG | 33413638 | Whole blood |
| EA | cg00574958 | 11 | 68607622 | *CPT1A* | -2.52E+00 | 2.50E-01 | 1.10E-23 | S-LDL-TG_pct | 33413638 | Whole blood |
| EA | cg00574958 | 11 | 68607622 | *CPT1A* | -1.80E+00 | 1.70E-01 | 2.10E-26 | S-VLDL-TG_pct | 33413638 | Whole blood |
| EA | cg00574958 | 11 | 68607622 | *CPT1A* | -9.50E-04 | 1.40E-04 | 1.90E-11 | age | 33450751 | Whole blood |
| EA | cg00574958 | 11 | 68607622 | *CPT1A* | -7.00E-03 | 1.25E-03 | 1.20E-08 | Type 2 diabetes | 33622391 | Whole blood |
| EA | cg00574958 | 11 | 68607622 | *CPT1A* | -1.82E-02 | 2.50E-03 | 2.80E-13 | Cardiovascular risk | 29326313 | Whole Blood |
| EA | cg09737197 | 11 | 68607675 | *CPT1A* | -1.62E-04 | 2.20E-05 | 5.03E-13 | Alcohol consumption per day | 27843151 | Whole blood |
| EA | cg09737197 | 11 | 68607675 | *CPT1A* | -9.30E-05 | 1.60E-05 | 4.19E-09 | Serum total cholesterol | 28213390 | Whole blood |
| EA | cg09737197 | 11 | 68607675 | *CPT1A* | -9.50E-05 | 1.60E-05 | 2.36E-09 | Serum total cholesterol | 28213390 | Whole blood |
| EA | cg09737197 | 11 | 68607675 | *CPT1A* | -9.10E-05 | 1.40E-05 | 4.25E-11 | Serum total cholesterol | 28213390 | Whole blood |
| EA | cg09737197 | 11 | 68607675 | *CPT1A* | -9.20E-05 | 1.40E-05 | 3.86E-11 | Serum total cholesterol | 28213390 | Whole blood |
| EA | cg09737197 | 11 | 68607675 | *CPT1A* | -7.15E-03 | 1.10E-03 | 9.13E-11 | Serum triglycerides | 28213390 | Whole blood |
| EA | cg09737197 | 11 | 68607675 | *CPT1A* | -7.03E-03 | 1.15E-03 | 1.01E-09 | Serum triglycerides | 28213390 | Whole blood |
| EA | cg09737197 | 11 | 68607675 | *CPT1A* | -8.04E-03 | 9.36E-04 | 9.29E-18 | Serum triglycerides | 28213390 | Whole blood |
| EA | cg09737197 | 11 | 68607675 | *CPT1A* | -7.90E-03 | 1.00E-03 | 7.23E-16 | Serum triglycerides | 28213390 | Whole blood |
| EA | cg09737197 | 11 | 68607675 | *CPT1A* | -1.04E-02 | 1.79E-03 | 6.18E-09 | Serum triglycerides | 28213390 | Whole blood |
| EA | cg09737197 | 11 | 68607675 | *CPT1A* | -1.02E-02 | 1.87E-03 | 4.58E-08 | Serum triglycerides | 28213390 | Whole blood |
| EA | cg09737197 | 11 | 68607675 | *CPT1A* | -1.42E-01 | 5.23E-03 | 7.90E-68 | Tissue | 30602389 | Buccal cells and peripheral blood mononuclear cells |
| EA | cg09737197 | 11 | 68607675 | *CPT1A* | NA | NA | 1.70E-12 | Alcohol consumption | 31789449 | Whole blood |
| EA | cg09737197 | 11 | 68607675 | *CPT1A* | NA | NA | 3.70E-11 | Alcohol consumption | 31789449 | Whole blood |
| EA | cg09737197 | 11 | 68607675 | *CPT1A* | NA | NA | 5.20E-13 | Alcohol consumption | 31789449 | Whole blood |
| EA | cg09737197 | 11 | 68607675 | *CPT1A* | 7.70E-04 | 9.20E-05 | 0 | age | 33450751 | Whole blood |
| EA | cg09737197 | 11 | 68607675 | *CPT1A* | 1.00E-03 | NA | 6.00E-46 | age | 33450751 | Whole blood |
| EA | cg17058475 | 11 | 68607737 | *CPT1A* | 4.39E-01 | 6.76E-02 | 8.65E-11 | Hypertriglyceridemic waist | 26798409 | Whole blood |
| EA | cg17058475 | 11 | 68607737 | *CPT1A* | -1.62E-04 | 2.30E-05 | 8.50E-13 | Alcohol consumption per day | 27843151 | Whole blood |
| EA | cg17058475 | 11 | 68607737 | *CPT1A* | -1.82E+01 | 2.40E+00 | 2.80E-12 | Body mass index | 28002404 | Whole blood |
| EA | cg17058475 | 11 | 68607737 | *CPT1A* | -1.25E+01 | 2.00E+00 | 9.60E-10 | Body mass index | 28002404 | Whole blood |
| EA | cg17058475 | 11 | 68607737 | *CPT1A* | NA | NA | 1.60E-20 | Body mass index | 28002404 | Whole blood |
| EA | cg17058475 | 11 | 68607737 | *CPT1A* | -1.27E+01 | 2.20E+00 | 7.90E-09 | Body mass index | 28002404 | Whole blood |
| EA | cg17058475 | 11 | 68607737 | *CPT1A* | -2.88E+01 | 3.95E+00 | 1.60E-10 | Body mass index | 28002404 | Whole blood |
| EA | cg17058475 | 11 | 68607737 | *CPT1A* | NA | NA | 2.28E-09 | Body mass index | 28095459 | Whole blood |
| EA | cg17058475 | 11 | 68607737 | *CPT1A* | -1.40E-02 | 2.00E-03 | 1.53E-12 | Triglycerides | 28194238 | Whole blood |
| EA | cg17058475 | 11 | 68607737 | *CPT1A* | -1.46E-02 | 2.00E-03 | 3.10E-13 | Triglycerides | 28194238 | Whole blood |
| EA | cg17058475 | 11 | 68607737 | *CPT1A* | -1.96E-02 | 2.40E-03 | 9.20E-16 | Triglycerides | 28194238 | Whole blood |
| EA | cg17058475 | 11 | 68607737 | *CPT1A* | -1.20E-02 | 2.10E-03 | 8.80E-09 | Triglycerides | 28194238 | Whole blood |
| EA | cg17058475 | 11 | 68607737 | *CPT1A* | -9.86E-03 | 1.00E-03 | 8.33E-23 | Serum triglycerides | 28213390 | Whole blood |
| EA | cg17058475 | 11 | 68607737 | *CPT1A* | -9.14E-03 | 1.05E-03 | 2.24E-18 | Serum triglycerides | 28213390 | Whole blood |
| EA | cg17058475 | 11 | 68607737 | *CPT1A* | -1.08E-02 | 8.57E-04 | 2.03E-36 | Serum triglycerides | 28213390 | Whole blood |
| EA | cg17058475 | 11 | 68607737 | *CPT1A* | -1.00E-02 | 9.00E-04 | 5.48E-29 | Serum triglycerides | 28213390 | Whole blood |
| EA | cg17058475 | 11 | 68607737 | *CPT1A* | -1.34E-02 | 1.67E-03 | 7.55E-16 | Serum triglycerides | 28213390 | Whole blood |
| EA | cg17058475 | 11 | 68607737 | *CPT1A* | -1.25E-02 | 1.75E-03 | 8.93E-13 | Serum triglycerides | 28213390 | Whole blood |
| EA | cg17058475 | 11 | 68607737 | *CPT1A* | -5.70E-05 | NA | 6.70E-09 | Systolic Blood Pressure | 29198723 | Whole blood, CD4+ T cells |
| EA | cg17058475 | 11 | 68607737 | *CPT1A* | -3.72E-01 | NA | 3.10E-09 | BMI | 29278407 | Whole blood |
| EA | cg17058475 | 11 | 68607737 | *CPT1A* | -1.78E+00 | 2.80E-01 | 2.10E-10 | fasting insulin | 31197173 | whole blood |
| EA | cg17058475 | 11 | 68607737 | *CPT1A* | NA | NA | 5.30E-17 | Alcohol consumption | 31789449 | Whole blood |
| EA | cg17058475 | 11 | 68607737 | *CPT1A* | NA | NA | 8.50E-18 | Alcohol consumption | 31789449 | Whole blood |
| EA | cg17058475 | 11 | 68607737 | *CPT1A* | NA | NA | 2.00E-22 | Alcohol consumption | 31789449 | Whole blood |
| EA | cg17058475 | 11 | 68607737 | *CPT1A* | 6.70E-04 | 1.20E-04 | 9.90E-09 | age | 33450751 | Whole blood |
| EA | cg17058475 | 11 | 68607737 | *CPT1A* | 1.10E-03 | NA | 2.00E-19 | age | 33450751 | Whole blood |
| EA | cg08657939 | 11 | 69295007 | *-* | 5.34E-01 | 8.20E-03 | 2.30E-133 | Tissue | 30602389 | Buccal cells and peripheral blood mononuclear cells |
| EA | cg19120513 | 11 | 102189303 | *BIRC3* | -5.90E-02 | 1.00E-02 | 8.00E-08 | Cancer treatment: Chest-RT and abdomen-RT | 33823916 | Whole blood |
| EA | cg10101263 | 11 | 130786633 | *SNX19* | 1.03E-02 | 1.40E-03 | 2.62E-13 | Rheumatoid arthritis | 23334450 | Whole blood |
| EA | cg10101263 | 11 | 130786633 | *SNX19* | -2.30E-04 | 3.30E-05 | 8.00E-12 | age | 33450751 | Whole blood |
| EA | cg12587729 | 12 | 50134921 | *TMBIM6* | 1.67E-02 | 2.30E-03 | 1.01E-12 | Rheumatoid arthritis | 23334450 | Whole blood |
| EA | cg12587729 | 12 | 50134921 | *TMBIM6* | -4.40E-04 | 6.80E-05 | 8.70E-11 | age | 33450751 | Whole blood |
| EA | cg17394331 | 12 | 68640287 | *-* | 1.57E-01 | 4.79E-03 | 7.10E-81 | Tissue | 30602389 | Buccal cells and peripheral blood mononuclear cells |
| EA | cg17394331 | 12 | 68640287 | *-* | -5.90E-04 | 6.70E-05 | 0 | age | 33450751 | Whole blood |
| EA | cg17394331 | 12 | 68640287 | *-* | 5.30E-04 | NA | 4.80E-09 | age | 33450751 | Whole blood |
| EA | cg20054394 | 12 | 132938338 | *-* | 1.89E-02 | 2.66E-03 | 2.40E-11 | Tissue | 30602389 | Buccal cells and peripheral blood mononuclear cells |
| EA | cg24327132 | 15 | 72520632 | *PKM2* | 3.26E-01 | 4.14E-03 | 1.40E-148 | Tissue | 30602389 | Buccal cells and peripheral blood mononuclear cells |
| EA | cg24327132 | 15 | 72520632 | *PKM2* | -8.92E-03 | 9.50E-04 | 8.80E-21 | Smoking | 31536415 | Whole blood, CD4+ T cells, CD14+ monocytes |
| EA | cg24327132 | 15 | 72520632 | *PKM2* | -2.70E-03 | 2.40E-04 | 0 | age | 33450751 | Whole blood |
| EA | cg24327132 | 15 | 72520632 | *PKM2* | 1.80E-02 | 1.60E-03 | 0 | age | 33450751 | Whole blood |
| EA | cg24327132 | 15 | 72520632 | *PKM2* | -7.70E-02 | 1.10E-02 | 3.60E-08 | Cancer treatment: chest-RT | 33823916 | Whole blood |
| EA | cg07878951 | 16 | 48176400 | *ABCC12* | -1.97E-02 | 2.45E-03 | 8.00E-14 | Tissue | 30602389 | Buccal cells and peripheral blood mononuclear cells |
| EA | cg07878951 | 16 | 48176400 | *ABCC12* | 1.60E-03 | NA | 4.90E-10 | age | 33450751 | Whole blood |
| EA | cg06375652 | 16 | 86100423 | *-* | 6.24E-02 | 2.43E-03 | 3.70E-64 | Tissue | 30602389 | Buccal cells and peripheral blood mononuclear cells |
| EA | cg06375652 | 16 | 86100423 | *-* | -9.40E-04 | 9.50E-05 | 0 | age | 33450751 | Whole blood |
| EA | cg23623659 | 17 | 4925475 | *KIF1C* | 2.40E-03 | NA | 2.20E-18 | age | 33450751 | Whole blood |
| EA | cg22165275 | 17 | 39382451 | *KRTAP9-2* | -1.90E-03 | 2.00E-04 | 0 | age | 33450751 | Whole blood |
| EA | cg22165275 | 17 | 39382451 | *KRTAP9-2* | 1.40E-03 | NA | 5.10E-09 | age | 33450751 | Whole blood |
| EA | cg22165275 | 17 | 39382451 | *KRTAP9-2* | 4.70E-03 | NA | 5.40E-08 | age | 33450751 | Whole blood |
| EA | cg03723356 | 17 | 46047760 | *CDK5RAP3* | 9.78E-02 | 4.57E-03 | 7.90E-53 | Tissue | 30602389 | Buccal cells and peripheral blood mononuclear cells |
| EA | cg03723356 | 17 | 46047760 | *CDK5RAP3* | -1.70E-03 | 2.00E-04 | 0 | age | 33450751 | Whole blood |
| EA | cg23475474 | 17 | 63527924 | *AXIN2* | 7.37E-01 | 8.02E-02 | 4.11E-20 | Ageing | 25888029 | Whole blood |
| EA | cg23475474 | 17 | 63527924 | *AXIN2* | 7.18E-04 | 1.32E-04 | 5.96E-08 | Gestational age | 27717397 | Cord blood |
| EA | cg23475474 | 17 | 63527924 | *AXIN2* | 2.79E-01 | 7.25E-03 | 2.30E-92 | Tissue | 30602389 | Buccal cells and peripheral blood mononuclear cells |
| EA | cg23475474 | 17 | 63527924 | *AXIN2* | -9.90E-04 | 1.80E-04 | 6.10E-08 | age | 33450751 | Whole blood |
| EA | cg23475474 | 17 | 63527924 | *AXIN2* | 1.40E-03 | NA | 5.00E-08 | age | 33450751 | Whole blood |
| EA | cg27295561 | 17 | 73754508 | *GALK1* | 5.51E-02 | 3.87E-03 | 6.80E-32 | Tissue | 30602389 | Buccal cells and peripheral blood mononuclear cells |
| EA | cg27295561 | 17 | 73754508 | *GALK1* | -7.00E-04 | 6.40E-05 | 0 | age | 33450751 | Whole blood |
| EA | cg10567810 | 18 | 11149068 | *FAM38B* | 5.98E-01 | 7.65E-02 | 5.20E-15 | Ageing | 25888029 | Whole blood |
| EA | cg10567810 | 18 | 11149068 | *FAM38B* | 3.81E-02 | 2.28E-03 | 2.90E-39 | Tissue | 30602389 | Buccal cells and peripheral blood mononuclear cells |
| EA | cg10567810 | 18 | 11149068 | *FAM38B* | 1.10E-03 | 8.80E-05 | 0 | age | 33450751 | Whole blood |
| EA | cg10567810 | 18 | 11149068 | *FAM38B* | 7.70E-04 | NA | 5.50E-20 | age | 33450751 | Whole blood |
| EA | cg13522114 | 19 | 6216405 | *MLLT1* | -2.35E+00 | 3.30E-01 | 1.10E-12 | HIV infection | 27105112 | Whole blood |
| EA | cg13522114 | 19 | 6216405 | *MLLT1* | 7.39E-02 | 2.79E-03 | 4.70E-66 | Tissue | 30602389 | Buccal cells and peripheral blood mononuclear cells |
| EA | cg13522114 | 19 | 6216405 | *MLLT1* | 8.90E-04 | NA | 4.90E-19 | age | 33450751 | Whole blood |
| EA | cg16740586 | 21 | 43655919 | *ABCG1* | 3.30E-02 | 5.00E-03 | 3.60E-11 | APOE e2 vs e4 | 33397400 | Whole blood |
| EA | cg18452324 | 22 | 37882397 | *MFNG* | -2.28E-02 | 2.14E-03 | 3.40E-21 | Tissue | 30602389 | Buccal cells and peripheral blood mononuclear cells |
| AA | cg16411101 | 1 | 54831653 | *SSBP3* | 9.82E-01 | 2.51E-01 | 9.25E-05 | HIV infection | 27105112 | Whole blood |
| AA | cg16411101 | 1 | 54831653 | *SSBP3* | -1.20E-03 | 1.10E-04 | 0 | age | 33450751 | Whole blood |
| AA | cg16411101 | 1 | 54831653 | *SSBP3* | 9.90E-04 | NA | 3.70E-19 | age | 33450751 | Whole blood |
| AA | cg16411101 | 1 | 54831653 | *SSBP3* | 2.90E-03 | NA | 1.70E-06 | age | 33450751 | Whole blood |
| AA | cg04348872 | 2 | 25141696 | *ADCY3* | 2.17E-02 | 1.95E-03 | 2.10E-22 | Tissue | 30602389 | Buccal cells and peripheral blood mononuclear cells |
| AA | cg04348872 | 2 | 25141696 | *ADCY3* | 1.10E-03 | NA | 2.30E-05 | age | 33450751 | Whole blood |
| AA | cg04348872 | 2 | 25141696 | *ADCY3* | -4.80E-03 | 1.20E-03 | 6.50E-05 | age | 33450751 | Whole blood |
| AA | cg26675329 | 2 | 103033753 | *IL18RAP* | 7.70E-03 | 1.50E-03 | 3.10E-07 | Smoking | 27651444 | CD4+ T cells, monocytes |
| AA | cg26675329 | 2 | 103033753 | *IL18RAP* | -3.98E+00 | 4.78E-01 | 9.54E-17 | HIV infection | 27105112 | Whole blood |
| AA | cg26675329 | 2 | 103033753 | *IL18RAP* | -6.18E-02 | 3.77E-03 | 2.20E-38 | Tissue | 30602389 | Buccal cells and peripheral blood mononuclear cells |
| AA | cg26675329 | 2 | 103033753 | *IL18RAP* | 7.10E-03 | 8.90E-04 | 1.40E-15 | Smoking | 31536415 | Whole blood, CD4+ T cells, CD14+ monocytes |
| AA | cg04747445 | 3 | 107241417 | *BBX* | NA | NA | 1.83E-25 | Age 4 vs age 0 | 28056824 | Whole blood, cord blood |
| AA | cg04747445 | 3 | 107241417 | *BBX* | NA | NA | 8.09E-05 | Tic disorders | 26499864 | Whole blood |
| AA | cg04747445 | 3 | 107241417 | *BBX* | 1.25E-02 | 2.00E-03 | 9.24E-10 | Rheumatoid arthritis | 23334450 | Whole blood |
| AA | cg04747445 | 3 | 107241417 | *BBX* | 5.78E-02 | 2.18E-03 | 3.00E-66 | Tissue | 30602389 | Buccal cells and peripheral blood mononuclear cells |
| AA | cg04747445 | 3 | 107241417 | *BBX* | -8.30E-04 | 9.20E-05 | 0 | age | 33450751 | Whole blood |
| AA | cg05416955 | 9 | 139265726 | *CARD9* | -1.97E+00 | 3.13E-01 | 2.89E-10 | HIV infection | 27105112 | Whole blood |
| AA | cg05416955 | 9 | 139265726 | *CARD9* | 2.17E-02 | 2.64E-03 | 2.60E-14 | Tissue | 30602389 | Buccal cells and peripheral blood mononuclear cells |
| AA | cg05416955 | 9 | 139265726 | *CARD9* | -4.60E-03 | 1.20E-03 | 8.70E-05 | age | 33450751 | Whole blood |
| AA | cg05416955 | 9 | 139265726 | *CARD9* | -9.10E-04 | 2.30E-04 | 6.10E-05 | Maternal body mass index | 33517419 | Cord blood |
| AA | cg23724016 | 11 | 617708 | *MUPCDH* | -6.90E-03 | 1.70E-03 | 6.20E-05 | Luteinizing hormone | NA | Whole blood |
| AA | cg23724016 | 11 | 617708 | *MUPCDH* | -1.41E-02 | 2.12E-03 | 2.90E-10 | Tissue | 30602389 | Buccal cells and peripheral blood mononuclear cells |
| AA | cg01111718 | 11 | 105762374 | *GRIA4* | 6.24E-02 | 3.96E-03 | 1.60E-36 | Tissue | 30602389 | Buccal cells and peripheral blood mononuclear cells |
| AA | cg21376908 | 17 | 55382424 | *MSI2* | -9.70E-04 | 2.00E-04 | 1.90E-06 | age | 33450751 | Whole blood |
| AA | cg21376908 | 17 | 55382424 | *MSI2* | 1.40E-03 | NA | 5.60E-09 | age | 33450751 | Whole blood |
| AA | cg21376908 | 17 | 55382424 | *MSI2* | 4.40E-03 | NA | 2.00E-05 | age | 33450751 | Whole blood |
| AA | cg21376908 | 17 | 55382424 | *MSI2* | -2.10E-03 | 4.70E-04 | 1.40E-05 | gestational age | 33564054 | Cord blood |
| AA | cg16197879 | 21 | 37839328 | *CLDN14* | -9.81E-03 | 2.03E-03 | 1.32E-06 | Schizophrenia | 27572077 | Whole blood |
| AA | cg16197879 | 21 | 37839328 | CLDN14 | -1.28E-02 | 2.10E-03 | 1.15E-09 | Rheumatoid arthritis | 23334450 | Whole blood |
| AA | cg16197879 | 21 | 37839328 | CLDN14 | -7.67E-03 | 1.59E-03 | 2.70E-06 | Tissue | 30602389 | Buccal cells and peripheral blood mononuclear cells |
| AA | cg16197879 | 21 | 37839328 | CLDN14 | -5.30E-04 | 9.60E-05 | 4.30E-08 | age | 33450751 | Whole blood |
| AA | cg16197879 | 21 | 37839328 | CLDN14 | 6.20E-04 | NA | 1.20E-06 | age | 33450751 | Whole blood |

Abbreviations: EA, survivors of European ancestry; AA, survivors of African ancestry.

Additional file 1: Table S7. The results among survivors of European ancestry in the current study for blood lipids-associated CpGs in the EWAS catalog based on previous studies in the general population (*P* < 9 x 10^-8^).

| CpG Labels | chr_hg38 | Start_hg38 | Gene HGNC | Lipid measurement | Exposure EWAS | | | Outcome EWAS | | |
| --- | --- | --- | --- | --- | --- | --- | --- | --- | --- | --- |
|  |  |  |  |  | Effect size | SE | P | Effect size | SE | P |
| cg17901584 | 1 | 54888032 | *DHCR24;RP11-67L3.4* | Triglycerides | -5.54E-05 | 6.53E-05 | 3.96E-01 | -1.60E+01 | 1.25E+01 | 2.03E-01 |
| cg03725309* | 1 | 109214962 | *SARS* |  | -5.76E-04 | 7.97E-05 | **1.23E-12** | -2.78E+01 | 1.04E+01 | 7.64E-03 |
| cg13925011 | 1 | 110673764 | *KCNA3* |  | -9.37E-05 | 3.82E-05 | 1.45E-02 | -1.76E+01 | 2.13E+01 | 4.10E-01 |
| cg16246545 | 1 | 119713317 | *PHGDH* |  | -1.76E-04 | 9.81E-05 | 7.27E-02 | -1.09E+01 | 9.22E+00 | 2.39E-01 |
| cg14476101 | 1 | 119713368 | *PHGDH* |  | -3.19E-04 | 1.15E-04 | 5.55E-03 | -9.87E+00 | 7.57E+00 | 1.93E-01 |
| cg19693031 | 1 | 145993515 | *TXNIP* |  | -5.29E-04 | 1.01E-04 | 2.08E-07 | -8.77E+00 | 8.47E+00 | 3.01E-01 |
| cg00948664 | 3 | 48897882 | *SLC25A20* |  | -2.99E-04 | 9.13E-05 | 1.11E-03 | -1.88E+01 | 9.72E+00 | 5.39E-02 |
| cg06690548 | 4 | 138241653 | *SLC7A11* |  | -4.08E-04 | 1.07E-04 | 1.50E-04 | -2.52E+01 | 8.19E+00 | 2.15E-03 |
| cg17501210 | 6 | 166556763 | *RPS6KA2* |  | -2.13E-04 | 8.62E-05 | 1.38E-02 | -1.18E+01 | 1.01E+01 | 2.45E-01 |
| cg21429551 | 7 | 30596145 | *AC005154.6;GARS* |  | -3.13E-04 | 1.28E-04 | 1.48E-02 | -1.33E+01 | 6.81E+00 | 5.10E-02 |
| cg03068497 | 7 | 30596221 | *AC005154.6;GARS* |  | -3.68E-04 | 1.23E-04 | 2.94E-03 | -9.70E+00 | 7.04E+00 | 1.68E-01 |
| cg19390658 | 7 | 30596559 | *GARS* |  | -1.53E-04 | 1.15E-04 | 1.85E-01 | -1.65E+01 | 7.45E+00 | 2.72E-02 |
| cg07504977 | 10 | 100371254 | *NA* |  | 3.55E-04 | 8.08E-05 | 1.29E-05 | 2.03E+01 | 1.09E+01 | 6.26E-02 |
| cg06734985 | 11 | 26827953 | *NA* |  | -1.07E-04 | 7.27E-05 | 1.41E-01 | -8.75E-01 | 1.17E+01 | 9.40E-01 |
| cg06178669 | 11 | 63567135 | *NA* |  | -5.26E-04 | 1.27E-04 | 3.93E-05 | -2.88E+00 | 6.91E+00 | 6.77E-01 |
| cg00574958* | 11 | 68840153 | *CPT1A* |  | -1.28E-03 | 1.20E-04 | **1.50E-24** | -3.29E+01 | 6.87E+00 | 1.83E-06 |
| cg09737197* | 11 | 68840206 | *CPT1A* |  | -6.41E-04 | 1.05E-04 | **1.60E-09** | -3.12E+01 | 8.06E+00 | 1.15E-04 |
| cg17058475* | 11 | 68840268 | *CPT1A* |  | -1.11E-03 | 1.31E-04 | **1.27E-16** | -3.33E+01 | 6.31E+00 | 1.61E-07 |
| cg01082498 | 11 | 68840756 | *CPT1A* |  | -6.31E-04 | 1.18E-04 | 1.24E-07 | -1.55E+01 | 7.20E+00 | 3.15E-02 |
| cg16618104 | 12 | 104459321 | *CHST11* |  | -1.83E-04 | 1.09E-04 | 9.35E-02 | -3.33E+00 | 7.92E+00 | 6.74E-01 |
| cg09213175 | 16 | 87768934 | *NA* |  | 1.66E-04 | 8.29E-05 | 4.55E-02 | 1.10E+01 | 1.06E+01 | 3.00E-01 |
| cg20544516 | 17 | 17813868 | *MIR33B;MIR6777;SREBF1* |  | 3.97E-05 | 5.43E-05 | 4.65E-01 | 1.40E+01 | 1.56E+01 | 3.72E-01 |
| cg08129017 | 17 | 17825345 | *SREBF1* |  | 1.05E-04 | 8.73E-05 | 2.29E-01 | 7.43E+00 | 1.00E+01 | 4.58E-01 |
| cg11024682 | 17 | 17826779 | *SREBF1* |  | 2.42E-04 | 5.15E-05 | 3.08E-06 | 3.58E+01 | 1.69E+01 | 3.39E-02 |
| cg08857797 | 17 | 42775680 | *VPS25* |  | 1.17E-04 | 7.14E-05 | 1.03E-01 | 2.08E+01 | 1.16E+01 | 7.23E-02 |
| cg24174557 | 17 | 59826182 | *VMP1* |  | -3.01E-04 | 1.02E-04 | 3.16E-03 | -8.28E+00 | 8.23E+00 | 3.15E-01 |
| cg22304262 | 19 | 46784520 | *SLC1A5* |  | -1.21E-04 | 6.32E-05 | 5.67E-02 | -1.42E+01 | 1.42E+01 | 3.16E-01 |
| cg27243685 | 21 | 42222255 | *ABCG1* |  | -3.21E-05 | 5.88E-05 | 5.85E-01 | -1.41E+00 | 1.40E+01 | 9.20E-01 |
| cg01881899 | 21 | 42232593 | *ABCG1* |  | 3.33E-04 | 8.74E-05 | 1.49E-04 | 1.80E+01 | 9.81E+00 | 6.73E-02 |
| cg01176028 | 21 | 42233123 | *ABCG1* |  | 9.48E-05 | 6.99E-05 | 1.76E-01 | 9.54E+00 | 1.22E+01 | 4.33E-01 |
| cg02370100 | 21 | 42235145 | *ABCG1* |  | 7.77E-05 | 7.14E-05 | 2.77E-01 | 4.76E+00 | 1.21E+01 | 6.95E-01 |
| cg17475467 | 1 | 54851095 | *DHCR24* | Total cholesterol | -2.73E-04 | 1.71E-04 | 1.10E-01 | -4.02E+00 | 4.79E+00 | 4.01E-01 |
| cg25536676 | 1 | 54887653 | *DHCR24;RP11-67L3.4;RP11-67L3.5* |  | 4.89E-04 | 1.73E-04 | 4.95E-03 | 6.18E+00 | 4.56E+00 | 1.76E-01 |
| cg17901584 | 1 | 54888032 | *DHCR24;RP11-67L3.4* |  | 2.88E-04 | 1.71E-04 | 9.27E-02 | 4.04E+00 | 4.50E+00 | 3.70E-01 |
| cg25738279 | 1 | 67561933 | *NA* |  | 1.79E-04 | 1.57E-04 | 2.54E-01 | 9.79E+00 | 5.28E+00 | 6.41E-02 |
| cg05337441 | 2 | 21043695 | *APOB* |  | 5.69E-04 | 5.20E-04 | 2.75E-01 | 1.87E+00 | 1.60E+00 | 2.42E-01 |
| cg23759710 | 2 | 42763816 | *OXER1* |  | -1.22E-04 | 1.02E-04 | 2.30E-01 | 7.02E+00 | 8.17E+00 | 3.90E-01 |
| cg06621027 | 2 | 79952632 | *CTNNA2* |  | -5.55E-04 | 4.23E-04 | 1.89E-01 | 2.78E-01 | 1.91E+00 | 8.84E-01 |
| cg21924447 | 2 | 137670232 | *THSD7B* |  | -2.10E-05 | 1.26E-04 | 8.67E-01 | 9.83E+00 | 6.40E+00 | 1.24E-01 |
| cg21550580 | 3 | 65769706 | *MAGI1* |  | 5.88E-05 | 1.36E-04 | 6.65E-01 | 1.19E+01 | 5.92E+00 | 4.47E-02 |
| cg23475018 | 3 | 124558790 | *KALRN* |  | 1.89E-04 | 1.33E-04 | 1.57E-01 | 7.10E+00 | 6.01E+00 | 2.38E-01 |
| cg05897122 | 4 | 52246540 | *NA* |  | 1.45E-04 | 2.13E-04 | 4.95E-01 | 8.95E+00 | 3.79E+00 | 1.85E-02 |
| cg22446998 | 5 | 156274808 | *SGCD* |  | 3.78E-05 | 1.88E-04 | 8.41E-01 | 1.43E+00 | 4.41E+00 | 7.45E-01 |
| cg15878619 | 6 | 30719595 | *TUBB* |  | 2.58E-04 | 2.30E-04 | 2.61E-01 | 6.65E+00 | 3.50E+00 | 5.76E-02 |
| cg09676013 | 6 | 33112722 | *HLA-DPB2* |  | -1.39E-04 | 1.68E-04 | 4.08E-01 | 1.30E+00 | 4.88E+00 | 7.90E-01 |
| cg21635307 | 6 | 89610157 | *ANKRD6;LYRM2* |  | -1.08E-04 | 2.11E-04 | 6.09E-01 | 7.59E+00 | 3.75E+00 | 4.32E-02 |
| cg13010621 | 6 | 91635004 | *CASC6* |  | 5.20E-05 | 9.92E-05 | 6.01E-01 | 5.01E+00 | 8.18E+00 | 5.40E-01 |
| cg18192491 | 6 | 114063706 | *HS3ST5;RP3-399L15.3* |  | 1.66E-04 | 1.18E-04 | 1.58E-01 | 1.98E+01 | 6.75E+00 | 3.46E-03 |
| cg03188976 | 7 | 134759112 | *CALD1* |  | 1.12E-04 | 2.32E-04 | 6.29E-01 | -1.55E+00 | 3.60E+00 | 6.67E-01 |
| cg00285394 | 8 | 124999711 | *SQLE* |  | 6.26E-04 | 3.27E-04 | 5.62E-02 | 3.99E+00 | 2.49E+00 | 1.10E-01 |
| cg14406814 | 9 | 10232875 | *PTPRD* |  | 1.08E-04 | 1.31E-04 | 4.13E-01 | 1.47E+00 | 6.20E+00 | 8.12E-01 |
| cg07121693 | 10 | 43652988 | *ZNF32-AS3* |  | 1.52E-05 | 1.77E-04 | 9.32E-01 | 8.73E+00 | 5.03E+00 | 8.27E-02 |
| cg09802018 | 11 | 46336011 | *DGKZ* |  | -1.55E-04 | 1.76E-04 | 3.80E-01 | -6.59E+00 | 4.52E+00 | 1.46E-01 |
| cg09737197 | 11 | 68840206 | *CPT1A* |  | -1.18E-03 | 2.79E-04 | 2.83E-05 | -6.57E+00 | 2.87E+00 | 2.23E-02 |
| cg15705971 | 11 | 92377095 | *FAT3* |  | 5.76E-05 | 1.24E-04 | 6.43E-01 | 8.93E+00 | 6.48E+00 | 1.68E-01 |
| cg22878054 | 11 | 99526520 | *CNTN5* |  | 2.41E-04 | 1.50E-04 | 1.07E-01 | 4.16E+00 | 5.65E+00 | 4.62E-01 |
| cg01575601 | 11 | 130529004 | *NA* |  | -9.41E-06 | 1.57E-04 | 9.52E-01 | 4.72E+00 | 5.30E+00 | 3.74E-01 |
| cg00252813 | 12 | 6533062 | *GAPDH;RP5-940J5.3* |  | 3.51E-04 | 1.49E-04 | 1.87E-02 | 9.36E+00 | 5.55E+00 | 9.18E-02 |
| cg17281677 | 12 | 6549390 | *IFFO1* |  | 3.70E-05 | 1.91E-04 | 8.47E-01 | 8.73E+00 | 4.23E+00 | 3.93E-02 |
| cg06844864 | 12 | 57456408 | *INHBE* |  | -2.20E-04 | 1.22E-04 | 7.31E-02 | -2.04E+00 | 5.95E+00 | 7.32E-01 |
| cg03308494 | 12 | 57619483 | *AC025165.8;SLC26A10* |  | -1.99E-04 | 1.68E-04 | 2.37E-01 | -1.68E+00 | 4.93E+00 | 7.33E-01 |
| cg01973483 | 12 | 123106575 | *PITPNM2* |  | -4.20E-04 | 1.94E-04 | 3.13E-02 | -4.15E+00 | 3.87E+00 | 2.83E-01 |
| cg20448706 | 14 | 32472634 | *AKAP6* |  | 1.62E-04 | 1.43E-04 | 2.57E-01 | 8.68E+00 | 5.90E+00 | 1.41E-01 |
| cg26874872 | 16 | 26145040 | *NA* |  | -1.45E-04 | 1.32E-04 | 2.75E-01 | 8.50E+00 | 6.28E+00 | 1.76E-01 |
| cg01955153 | 16 | 50735940 | *RP11-327F22.2* |  | 2.23E-04 | 1.36E-04 | 1.02E-01 | 1.11E+01 | 5.85E+00 | 5.90E-02 |
| cg07839457 | 16 | 56989109 | *NLRC5* |  | 7.96E-04 | 3.73E-04 | 3.31E-02 | 1.90E+00 | 2.37E+00 | 4.23E-01 |
| cg15380836 | 17 | 1650046 | *RILP* |  | 2.81E-04 | 1.31E-04 | 3.28E-02 | 4.77E+00 | 6.30E+00 | 4.48E-01 |
| cg10001987 | 17 | 7478449 | *ZBTB4* |  | 2.72E-04 | 3.51E-04 | 4.40E-01 | -1.12E-01 | 2.28E+00 | 9.61E-01 |
| cg20637199 | 19 | 1226534 | *STK11* |  | -4.86E-04 | 4.00E-04 | 2.24E-01 | 1.49E-01 | 1.95E+00 | 9.39E-01 |
| cg22023664 | 19 | 2619609 | *GNG7* |  | 9.27E-05 | 1.38E-04 | 5.02E-01 | -5.14E+00 | 7.25E+00 | 4.78E-01 |
| cg16466652 | 19 | 6271948 | *MLLT1* |  | -7.46E-05 | 1.20E-04 | 5.33E-01 | -4.23E-01 | 6.81E+00 | 9.51E-01 |
| cg09978077 | 22 | 41833978 | *RP5-821D11.7;SREBF2* |  | 6.40E-04 | 2.00E-04 | 1.45E-03 | -1.50E+00 | 3.98E+00 | 7.06E-01 |
| cg16000331 | 22 | 41834133 | *RP5-821D11.7;SREBF2* |  | 5.67E-04 | 2.06E-04 | 5.94E-03 | 1.78E+00 | 3.82E+00 | 6.41E-01 |
| cg25402049 | 1 | 13703379 | *PRDM2* | High-density lipoprotein | 6.51E-04 | 7.39E-04 | 3.78E-01 | 1.18E-01 | 8.79E-01 | 8.93E-01 |
| cg13876650 | 1 | 25819513 | *MTFR1L;RP1-317E23.3;RP1-317E23.6* |  | 5.55E-05 | 3.16E-04 | 8.61E-01 | -2.45E-01 | 2.06E+00 | 9.05E-01 |
| cg25130381 | 1 | 27114229 | *SLC9A1* |  | -1.06E-03 | 4.80E-04 | 2.74E-02 | -1.20E-01 | 1.12E+00 | 9.15E-01 |
| cg12728588 | 1 | 35559887 | *NCDN* |  | -1.26E-03 | 3.77E-04 | 8.74E-04 | -2.93E+00 | 1.57E+00 | 6.26E-02 |
| cg17901584 | 1 | 54888032 | *DHCR24;RP11-67L3.4* |  | 1.35E-03 | 4.47E-04 | 2.56E-03 | -3.70E-01 | 1.34E+00 | 7.83E-01 |
| cg24694018 | 1 | 145977464 | *CH17-270A2.2;POLR3GL* |  | 9.81E-04 | 3.53E-04 | 5.60E-03 | -1.37E+00 | 1.67E+00 | 4.12E-01 |
| cg24678869 | 1 | 153947161 | *DENND4B* |  | -1.29E-03 | 3.48E-04 | 2.29E-04 | -1.62E+00 | 1.69E+00 | 3.37E-01 |
| cg25217710 | 1 | 156639730 | *RP11-284F21.9* |  | 5.24E-06 | 3.27E-04 | 9.87E-01 | -6.69E-01 | 1.94E+00 | 7.31E-01 |
| cg25739016 | 1 | 167669680 | *RCSD1* |  | -4.70E-04 | 4.56E-04 | 3.03E-01 | -1.91E-01 | 1.39E+00 | 8.91E-01 |
| cg13222915 | 1 | 184629459 | *NA* |  | -1.26E-04 | 3.70E-04 | 7.32E-01 | -3.20E+00 | 1.73E+00 | 6.43E-02 |
| cg00567854 | 1 | 203304564 | *BTG2;LINC01136* |  | -1.42E-03 | 5.76E-04 | 1.36E-02 | -8.64E-01 | 1.06E+00 | 4.14E-01 |
| cg10717869 | 1 | 205811783 | *SLC41A1* |  | -1.06E-03 | 4.20E-04 | 1.17E-02 | -9.47E-01 | 1.44E+00 | 5.10E-01 |
| cg19638572 | 1 | 206559810 | *RASSF5* |  | 1.41E-04 | 4.72E-04 | 7.66E-01 | 2.03E+00 | 1.28E+00 | 1.12E-01 |
| cg01101459 | 1 | 234735729 | *NA* |  | -3.99E-04 | 4.43E-04 | 3.69E-01 | 1.46E+00 | 1.44E+00 | 3.13E-01 |
| cg13139542 | 2 | 8102684 | *LINC00298;LINC00299* |  | 7.51E-04 | 5.98E-04 | 2.10E-01 | 1.04E+00 | 1.08E+00 | 3.38E-01 |
| cg04061506 | 2 | 47910121 | *NA* |  | -3.07E-03 | 6.24E-04 | 1.10E-06 | 6.86E-02 | 1.01E+00 | 9.46E-01 |
| cg02717339 | 2 | 96859029 | *ANKRD23;ANKRD39* |  | -1.15E-03 | 6.17E-04 | 6.29E-02 | -2.16E+00 | 1.05E+00 | 4.06E-02 |
| ch.2.101965435R | 2 | 101982540 | *LINC01127* |  | 9.78E-04 | 7.21E-04 | 1.75E-01 | 1.71E-01 | 9.12E-01 | 8.51E-01 |
| cg05295703 | 2 | 102279251 | *NA* |  | -4.80E-04 | 3.58E-04 | 1.80E-01 | -3.59E+00 | 1.70E+00 | 3.48E-02 |
| cg06876354 | 2 | 120262612 | *RALB* |  | -1.42E-03 | 2.94E-04 | 1.62E-06 | -4.72E+00 | 2.05E+00 | 2.17E-02 |
| cg03339817 | 2 | 127700706 | *SFT2D3* |  | 1.36E-04 | 5.46E-04 | 8.04E-01 | -3.08E+00 | 1.21E+00 | 1.08E-02 |
| cg25889918 | 2 | 179051456 | *CCDC141* |  | -5.58E-04 | 3.79E-04 | 1.41E-01 | 1.29E+00 | 1.64E+00 | 4.31E-01 |
| cg09613192 | 2 | 180523810 | *NA* |  | -1.09E-03 | 4.60E-04 | 1.80E-02 | 1.56E-01 | 1.40E+00 | 9.11E-01 |
| cg19351166 | 2 | 208268907 | *PIKFYVE* |  | -4.78E-04 | 4.13E-04 | 2.47E-01 | 9.92E-01 | 1.56E+00 | 5.25E-01 |
| cg04262505 | 2 | 216985899 | *AC007563.5* |  | -1.01E-03 | 3.72E-04 | 7.02E-03 | -4.51E-01 | 1.75E+00 | 7.97E-01 |
| cg12315466 | 2 | 233059103 | *INPP5D* |  | -1.42E-04 | 2.83E-04 | 6.15E-01 | 4.30E+00 | 2.28E+00 | 5.96E-02 |
| cg07691624 | 3 | 9844513 | *RPUSD3;TTLL3* |  | 8.46E-05 | 4.67E-04 | 8.56E-01 | 2.36E+00 | 1.42E+00 | 9.69E-02 |
| cg22257056 | 3 | 32421273 | *CMTM7* |  | -6.44E-04 | 2.87E-04 | 2.49E-02 | -1.85E+00 | 2.10E+00 | 3.80E-01 |
| cg20732160 | 3 | 48552606 | *PFKFB4* |  | -8.27E-04 | 3.60E-04 | 2.20E-02 | 1.03E+00 | 1.75E+00 | 5.58E-01 |
| cg00108715 | 3 | 52530998 | *NT5DC2* |  | -4.46E-04 | 3.69E-04 | 2.27E-01 | -1.05E-01 | 1.68E+00 | 9.50E-01 |
| cg00959259 | 3 | 122563127 | *DTX3L;PARP9* |  | 1.67E-03 | 9.84E-04 | 9.07E-02 | 2.05E-01 | 6.76E-01 | 7.62E-01 |
| cg02578470 | 3 | 127601826 | *MCM2* |  | -5.30E-04 | 3.89E-04 | 1.73E-01 | -4.60E-01 | 1.63E+00 | 7.79E-01 |
| cg07730360 | 3 | 129126782 | *ISY1-RAB43* |  | 1.08E-04 | 4.47E-04 | 8.09E-01 | 4.09E-01 | 1.48E+00 | 7.82E-01 |
| cg14468090 | 3 | 129314020 | *H1FX-AS1* |  | -1.41E-03 | 4.65E-04 | 2.53E-03 | -7.07E-01 | 1.44E+00 | 6.23E-01 |
| cg12825509 | 3 | 185930779 | *TRA2B* |  | 2.39E-04 | 4.52E-04 | 5.97E-01 | 1.26E+00 | 1.48E+00 | 3.91E-01 |
| cg26943120 | 4 | 5470388 | *STK32B* |  | 1.27E-03 | 9.83E-04 | 1.96E-01 | 2.66E-01 | 6.54E-01 | 6.84E-01 |
| cg08174890 | 4 | 9556455 | *MIR548I2* |  | -1.07E-04 | 4.59E-04 | 8.16E-01 | -6.58E-01 | 1.30E+00 | 6.14E-01 |
| cg06151145 | 4 | 48344416 | *SLAIN2* |  | -1.87E-03 | 4.64E-04 | 6.35E-05 | -4.41E-01 | 1.36E+00 | 7.47E-01 |
| cg10179300 | 5 | 14147508 | *TRIO* |  | -2.96E-04 | 6.63E-04 | 6.55E-01 | -4.58E-01 | 9.49E-01 | 6.29E-01 |
| cg00819078 | 5 | 16614971 | *RETREG1;RP11-260E18.1* |  | -2.25E-03 | 6.84E-04 | 1.07E-03 | -1.00E+00 | 9.28E-01 | 2.80E-01 |
| cg19742105 | 5 | 89130611 | *MEF2C-AS1* |  | 1.24E-03 | 4.12E-04 | 2.79E-03 | 3.35E-01 | 1.61E+00 | 8.36E-01 |
| cg18034719 | 5 | 177433861 | *GRK6* |  | 3.05E-04 | 4.74E-04 | 5.20E-01 | 1.14E+00 | 1.29E+00 | 3.78E-01 |
| cg20605134 | 6 | 15400230 | *JARID2* |  | -4.85E-04 | 2.89E-04 | 9.31E-02 | -6.08E-02 | 2.26E+00 | 9.79E-01 |
| cg26405097 | 6 | 15428069 | *JARID2* |  | -1.30E-03 | 3.94E-04 | 1.00E-03 | -2.74E+00 | 1.69E+00 | 1.05E-01 |
| cg22753611 | 6 | 17472660 | *CAP2* |  | 2.65E-04 | 5.14E-04 | 6.06E-01 | 1.25E+00 | 1.28E+00 | 3.29E-01 |
| cg13522406 | 6 | 30614042 | *PPP1R10* |  | -5.77E-04 | 4.71E-04 | 2.21E-01 | 2.03E+00 | 1.41E+00 | 1.49E-01 |
| cg14352682 | 6 | 30646390 | *ATAT1;C6orf136* |  | -2.81E-04 | 3.25E-04 | 3.87E-01 | -3.05E+00 | 1.92E+00 | 1.11E-01 |
| cg16609995 | 6 | 32191258 | *GPSM3;PBX2* |  | -8.70E-04 | 3.53E-04 | 1.40E-02 | -2.43E+00 | 1.86E+00 | 1.93E-01 |
| cg09572125 | 6 | 33432699 | *SYNGAP1* |  | -2.22E-04 | 3.26E-04 | 4.97E-01 | 2.54E-01 | 1.80E+00 | 8.87E-01 |
| cg12269535 | 6 | 43174275 | *SRF* |  | 4.83E-04 | 5.26E-04 | 3.59E-01 | 5.63E-01 | 1.29E+00 | 6.62E-01 |
| cg06560379 | 6 | 44263567 | *NFKBIE* |  | 1.30E-03 | 5.39E-04 | 1.66E-02 | 1.13E+00 | 1.17E+00 | 3.36E-01 |
| cg09473207 | 6 | 154758318 | *SCAF8* |  | -9.71E-04 | 3.85E-04 | 1.18E-02 | -1.25E+00 | 1.52E+00 | 4.10E-01 |
| cg17501210 | 6 | 166556763 | *RPS6KA2* |  | 1.25E-03 | 5.95E-04 | 3.67E-02 | 1.09E+00 | 1.08E+00 | 3.15E-01 |
| cg24939194 | 7 | 2523976 | *LFNG* |  | -9.21E-04 | 3.40E-04 | 6.97E-03 | -3.81E-01 | 1.80E+00 | 8.32E-01 |
| cg03676485 | 7 | 2524181 | *LFNG* |  | -1.01E-03 | 4.26E-04 | 1.81E-02 | 5.11E-01 | 1.44E+00 | 7.23E-01 |
| cg26804423 | 7 | 8161503 | *ICA1* |  | -6.81E-04 | 4.38E-04 | 1.21E-01 | -1.02E+00 | 1.44E+00 | 4.79E-01 |
| cg02902261 | 7 | 73576819 | *TBL2* |  | 1.85E-04 | 4.60E-04 | 6.88E-01 | 7.94E-01 | 1.36E+00 | 5.58E-01 |
| cg04577162 | 7 | 74253066 | *RFC2* |  | 5.90E-05 | 3.61E-04 | 8.70E-01 | -1.14E+00 | 1.71E+00 | 5.05E-01 |
| cg01676795 | 7 | 75957029 | *POR* |  | -3.96E-04 | 5.61E-04 | 4.80E-01 | 7.07E-01 | 1.16E+00 | 5.41E-01 |
| cg20782117 | 7 | 96236318 | *SLC25A13* |  | 3.06E-04 | 4.36E-04 | 4.83E-01 | 7.37E-01 | 1.48E+00 | 6.20E-01 |
| cg10127660 | 8 | 22138720 | *REEP4* |  | -2.62E-04 | 3.69E-04 | 4.78E-01 | -1.55E+00 | 1.77E+00 | 3.80E-01 |
| cg13821176 | 8 | 125436095 | *TRIB1* |  | -8.49E-04 | 5.20E-04 | 1.03E-01 | 1.18E-01 | 1.18E+00 | 9.20E-01 |
| cg08788930 | 8 | 141191585 | *DENND3* |  | 4.05E-05 | 6.74E-04 | 9.52E-01 | 1.78E+00 | 9.58E-01 | 6.39E-02 |
| cg15551881 | 9 | 120926436 | *TRAF1* |  | -9.71E-04 | 5.37E-04 | 7.10E-02 | -1.84E+00 | 1.14E+00 | 1.07E-01 |
| cg13622265 | 9 | 121822194 | *TTLL11* |  | 5.42E-04 | 9.76E-04 | 5.79E-01 | 2.49E-01 | 7.38E-01 | 7.36E-01 |
| cg06372475 | 10 | 71774528 | *CDH23;VSIR* |  | -4.17E-04 | 3.46E-04 | 2.29E-01 | 2.12E-01 | 1.92E+00 | 9.12E-01 |
| cg17485681 | 10 | 71805867 | *CDH23* |  | -1.76E-03 | 1.06E-03 | 9.66E-02 | -1.53E+00 | 6.26E-01 | 1.44E-02 |
| cg26002370 | 10 | 71837099 | *PSAP* |  | -1.26E-03 | 5.52E-04 | 2.27E-02 | -2.46E+00 | 1.13E+00 | 2.99E-02 |
| cg16407699 | 10 | 72260669 | *NA* |  | -1.41E-03 | 8.64E-04 | 1.03E-01 | 1.06E-01 | 7.42E-01 | 8.86E-01 |
| cg11849692 | 10 | 102116211 | *LDB1* |  | -1.67E-03 | 4.51E-04 | 2.20E-04 | -1.67E+00 | 1.41E+00 | 2.35E-01 |
| cg14939082 | 10 | 102776232 | *WBP1L* |  | -9.80E-04 | 3.20E-04 | 2.25E-03 | 2.04E-01 | 2.03E+00 | 9.20E-01 |
| cg26640901 | 10 | 102776277 | *WBP1L* |  | -1.03E-03 | 3.53E-04 | 3.62E-03 | -9.62E-01 | 1.46E+00 | 5.10E-01 |
| cg05457221 | 10 | 132458932 | *NA* |  | -1.20E-03 | 4.48E-04 | 7.83E-03 | -9.35E-01 | 1.46E+00 | 5.23E-01 |
| cg00805360 | 10 | 133277705 | *ADAM8* |  | -7.78E-04 | 3.89E-04 | 4.61E-02 | 7.17E-02 | 1.61E+00 | 9.65E-01 |
| cg26446803 | 11 | 1786001 | *NA* |  | -1.14E-04 | 3.84E-04 | 7.66E-01 | -1.62E+00 | 1.65E+00 | 3.26E-01 |
| cg04548926 | 11 | 2197822 | *NA* |  | -5.67E-04 | 3.19E-04 | 7.62E-02 | -7.15E-01 | 1.97E+00 | 7.17E-01 |
| cg14816825 | 11 | 12106655 | *MICAL2* |  | -6.07E-04 | 4.98E-04 | 2.24E-01 | 1.16E+00 | 1.28E+00 | 3.65E-01 |
| cg02855207 | 11 | 73375970 | *RELT* |  | -7.36E-04 | 3.36E-04 | 2.88E-02 | 9.80E-01 | 1.77E+00 | 5.81E-01 |
| cg09777883 | 11 | 112222972 | *BCO2* |  | -8.76E-05 | 4.34E-04 | 8.40E-01 | 4.28E-01 | 1.53E+00 | 7.79E-01 |
| cg24065451 | 11 | 128549616 | *ETS1* |  | -1.36E-03 | 4.45E-04 | 2.25E-03 | 8.72E-01 | 1.37E+00 | 5.24E-01 |
| cg04181189 | 11 | 129947522 | *PRDM10* |  | -9.83E-04 | 3.82E-04 | 1.02E-02 | -5.36E-01 | 1.59E+00 | 7.36E-01 |
| cg22488164 | 12 | 14563975 | *PLBD1* |  | -4.19E-04 | 4.81E-04 | 3.85E-01 | -1.76E+00 | 1.38E+00 | 2.03E-01 |
| cg18805734 | 12 | 31300966 | *SINHCAF* |  | -5.36E-04 | 3.12E-04 | 8.63E-02 | -4.41E-02 | 1.80E+00 | 9.80E-01 |
| cg01856529 | 12 | 54259306 | *CBX5* |  | 2.17E-05 | 4.60E-04 | 9.62E-01 | 2.92E+00 | 1.35E+00 | 3.13E-02 |
| cg03074946 | 12 | 120249890 | *PXN* |  | -1.50E-03 | 4.76E-04 | 1.75E-03 | -3.44E-01 | 1.33E+00 | 7.96E-01 |
| cg21042285 | 13 | 23380839 | *SACS* |  | -6.72E-04 | 6.99E-04 | 3.36E-01 | -1.07E+00 | 9.29E-01 | 2.49E-01 |
| cg19750657 | 13 | 38361829 | *UFM1* |  | -9.35E-04 | 5.39E-04 | 8.34E-02 | -1.31E+00 | 1.22E+00 | 2.83E-01 |
| cg26851608 | 13 | 51804343 | *DHRS12;RP11-327P2.5* |  | 2.22E-04 | 8.70E-04 | 7.98E-01 | 1.29E+00 | 6.98E-01 | 6.54E-02 |
| cg19881557 | 14 | 20499266 | *NA* |  | -1.00E-05 | 3.00E-04 | 9.73E-01 | -1.93E+00 | 2.02E+00 | 3.40E-01 |
| cg15821562 | 14 | 89616930 | *FOXN3;RP11-33N16.3* |  | -3.12E-04 | 4.46E-04 | 4.84E-01 | -5.12E-01 | 1.34E+00 | 7.03E-01 |
| cg21113318 | 14 | 92517300 | *RIN3* |  | -8.12E-04 | 3.57E-04 | 2.31E-02 | -3.82E+00 | 1.55E+00 | 1.41E-02 |
| cg27394566 | 14 | 104925297 | *PLD4* |  | -3.89E-04 | 3.03E-04 | 1.99E-01 | 1.24E+00 | 2.02E+00 | 5.41E-01 |
| cg15483436 | 14 | 105390870 | *PACS2* |  | -1.36E-04 | 4.16E-04 | 7.43E-01 | 2.08E+00 | 1.52E+00 | 1.70E-01 |
| cg07814318 | 15 | 31332380 | *KLF13* |  | -1.47E-03 | 8.70E-04 | 9.26E-02 | -4.17E-01 | 7.47E-01 | 5.77E-01 |
| cg06192883 | 15 | 52261973 | *MYO5C* |  | -1.68E-03 | 5.52E-04 | 2.48E-03 | -5.02E-01 | 1.16E+00 | 6.66E-01 |
| cg14473904 | 15 | 92921856 | *CHD2;RP11-437B10.1* |  | -1.30E-03 | 4.25E-04 | 2.25E-03 | -1.44E+00 | 1.43E+00 | 3.16E-01 |
| cg24002003 | 15 | 101127937 | *NA* |  | 9.84E-04 | 3.82E-04 | 1.02E-02 | -1.32E+00 | 1.66E+00 | 4.25E-01 |
| cg09581649 | 16 | 4522733 | *CDIP1* |  | -7.33E-04 | 3.11E-04 | 1.86E-02 | -1.02E+00 | 2.07E+00 | 6.24E-01 |
| cg07175797 | 16 | 50283744 | *ADCY7* |  | -5.50E-04 | 3.21E-04 | 8.74E-02 | -1.17E+00 | 1.98E+00 | 5.53E-01 |
| cg02879453 | 16 | 50287906 | *ADCY7* |  | 1.45E-04 | 4.66E-04 | 7.55E-01 | -2.86E+00 | 1.33E+00 | 3.16E-02 |
| cg23580000 | 16 | 50288244 | *ADCY7* |  | -3.48E-04 | 4.11E-04 | 3.97E-01 | -6.02E-01 | 1.56E+00 | 7.00E-01 |
| cg03031932 | 16 | 81513532 | *CMIP* |  | -4.63E-04 | 3.42E-04 | 1.76E-01 | -3.92E+00 | 1.86E+00 | 3.50E-02 |
| cg03014634 | 16 | 85171423 | *CTC-786C10.1* |  | -2.65E-04 | 3.23E-04 | 4.13E-01 | 4.40E+00 | 1.92E+00 | 2.23E-02 |
| cg03699074 | 16 | 88783466 | *PIEZO1* |  | -1.44E-04 | 7.96E-04 | 8.56E-01 | -3.48E-01 | 7.90E-01 | 6.60E-01 |
| cg10183965 | 17 | 3921202 | *NA* |  | -3.78E-04 | 3.34E-04 | 2.59E-01 | 1.88E+00 | 1.96E+00 | 3.36E-01 |
| cg07565956 | 17 | 7477968 | *ZBTB4* |  | -3.76E-04 | 5.82E-04 | 5.19E-01 | 3.06E-01 | 1.03E+00 | 7.67E-01 |
| cg14633721 | 17 | 8966546 | *CTB-41I6.1;CTB-41I6.2;PIK3R5* |  | -6.46E-04 | 3.66E-04 | 7.82E-02 | -1.10E+00 | 1.75E+00 | 5.29E-01 |
| cg11024682 | 17 | 17826779 | *SREBF1* |  | -9.03E-04 | 3.59E-04 | 1.21E-02 | -4.39E+00 | 1.78E+00 | 1.36E-02 |
| cg16611584 | 17 | 19905764 | *AKAP10* |  | -2.98E-04 | 8.51E-04 | 7.26E-01 | 8.89E-01 | 6.73E-01 | 1.87E-01 |
| cg12866859 | 17 | 45146964 | *AC142472.6* |  | -4.65E-04 | 3.27E-04 | 1.56E-01 | -9.88E-01 | 1.90E+00 | 6.03E-01 |
| cg01130991 | 17 | 48433029 | *NA* |  | -1.20E-03 | 6.84E-04 | 7.94E-02 | 8.11E-01 | 9.66E-01 | 4.01E-01 |
| cg02650017 | 17 | 49224251 | *PHOSPHO1* |  | 3.51E-04 | 4.70E-04 | 4.55E-01 | 1.39E+00 | 1.39E+00 | 3.19E-01 |
| cg09199486 | 17 | 50182086 | *HILS1* |  | -4.08E-04 | 2.44E-04 | 9.46E-02 | -1.00E+00 | 2.54E+00 | 6.93E-01 |
| cg08352115 | 17 | 68359915 | *ARSG* |  | 4.90E-04 | 4.37E-04 | 2.63E-01 | -5.49E-01 | 1.45E+00 | 7.05E-01 |
| cg27366162 | 17 | 68379053 | *ARSG* |  | 5.65E-05 | 3.63E-04 | 8.76E-01 | 6.35E-01 | 1.71E+00 | 7.11E-01 |
| cg02797539 | 17 | 74744384 | *RAB37* |  | -4.17E-05 | 3.42E-04 | 9.03E-01 | 5.28E-02 | 1.88E+00 | 9.78E-01 |
| cg21486834 | 17 | 76481459 | *RHBDF2* |  | -7.19E-04 | 4.17E-04 | 8.47E-02 | -9.63E-01 | 1.46E+00 | 5.10E-01 |
| cg05264101 | 17 | 77884225 | *FLJ45079* |  | -1.56E-03 | 4.27E-04 | 2.76E-04 | -1.19E+00 | 1.46E+00 | 4.17E-01 |
| cg06710464 | 17 | 81073894 | *BAIAP2* |  | -3.33E-05 | 3.72E-04 | 9.29E-01 | -2.40E-01 | 1.65E+00 | 8.85E-01 |
| cg11969813 | 17 | 81858682 | *P4HB* |  | -8.55E-05 | 6.45E-04 | 8.94E-01 | -4.72E-01 | 9.78E-01 | 6.29E-01 |
| cg01944226 | 17 | 82230179 | *SLC16A3* |  | 1.17E-04 | 4.97E-04 | 8.14E-01 | 1.43E+00 | 1.33E+00 | 2.82E-01 |
| cg25877299 | 17 | 82315413 | *CD7* |  | -5.23E-04 | 3.71E-04 | 1.59E-01 | -2.92E-01 | 1.65E+00 | 8.60E-01 |
| cg17992670 | 19 | 11105629 | *LDLR* |  | -7.11E-04 | 4.89E-04 | 1.47E-01 | -9.46E-01 | 1.34E+00 | 4.82E-01 |
| cg26313301 | 19 | 11108938 | *LDLR* |  | -4.39E-04 | 5.34E-04 | 4.11E-01 | 1.16E-01 | 1.17E+00 | 9.21E-01 |
| cg01751802 | 19 | 11198962 | *KANK2* |  | -7.99E-05 | 5.74E-04 | 8.89E-01 | 1.60E+00 | 1.12E+00 | 1.52E-01 |
| cg16655795 | 19 | 42393085 | *NA* |  | -6.73E-04 | 3.42E-04 | 4.93E-02 | 1.18E+00 | 1.93E+00 | 5.42E-01 |
| cg25667998 | 19 | 56194063 | *ZSCAN5B* |  | 5.49E-04 | 5.45E-04 | 3.14E-01 | 7.38E-02 | 1.17E+00 | 9.50E-01 |
| cg18952506 | 20 | 19935442 | *RIN2* |  | -6.02E-04 | 4.49E-04 | 1.80E-01 | -1.74E+00 | 1.37E+00 | 2.04E-01 |
| cg27243685 | 21 | 42222255 | *ABCG1* |  | -2.09E-04 | 4.06E-04 | 6.06E-01 | -8.37E-01 | 1.49E+00 | 5.75E-01 |
| cg25739715 | 22 | 30267891 | *OSM* |  | -2.86E-04 | 5.09E-04 | 5.75E-01 | -7.98E-01 | 1.26E+00 | 5.28E-01 |
| cg04934530 | 22 | 46684273 | *NA* |  | -1.09E-03 | 5.96E-04 | 6.89E-02 | -6.02E-01 | 1.08E+00 | 5.78E-01 |
| cg00156001 | 22 | 49801376 | *BRD1* |  | -1.70E-04 | 7.16E-04 | 8.13E-01 | 5.39E-01 | 8.79E-01 | 5.40E-01 |
| cg25551219 | 22 | 50471435 | *SBF1* |  | -3.89E-04 | 3.66E-04 | 2.88E-01 | -2.76E+00 | 1.75E+00 | 1.16E-01 |
| cg16736826 | 1 | 41485840 | *EDN2* | Low-density lipoprotein | -6.94E-05 | 1.38E-04 | 6.16E-01 | 1.48E+01 | 5.18E+00 | 4.37E-03 |
| cg10587082 | 1 | 208120132 | *PLXNA2* |  | 1.21E-04 | 1.69E-04 | 4.71E-01 | 5.68E+00 | 3.99E+00 | 1.55E-01 |
| cg13359998 | 1 | 230106016 | *GALNT2* |  | 3.72E-04 | 1.62E-04 | 2.20E-02 | -1.54E+00 | 4.34E+00 | 7.22E-01 |
| cg05337441 | 2 | 21043695 | *APOB* |  | 1.12E-03 | 6.16E-04 | 6.83E-02 | 8.44E-01 | 1.19E+00 | 4.80E-01 |
| cg25073563 | 2 | 40287370 | *SLC8A1* |  | -2.36E-04 | 1.81E-04 | 1.92E-01 | 1.58E+00 | 3.98E+00 | 6.92E-01 |
| cg23759710 | 2 | 42763816 | *OXER1* |  | -2.51E-05 | 1.20E-04 | 8.34E-01 | 1.14E+00 | 6.12E+00 | 8.52E-01 |
| cg06621027 | 2 | 79952632 | *CTNNA2* |  | -6.89E-04 | 5.03E-04 | 1.71E-01 | 1.92E-01 | 1.43E+00 | 8.93E-01 |
| cg02115781 | 2 | 171318762 | *METTL8* |  | 2.80E-04 | 1.85E-04 | 1.29E-01 | 3.89E+00 | 3.79E+00 | 3.06E-01 |
| cg21550580 | 3 | 65769706 | *MAGI1* |  | 4.70E-05 | 1.59E-04 | 7.68E-01 | 9.26E+00 | 4.48E+00 | 3.89E-02 |
| cg25436634 | 3 | 181327481 | *SOX2-OT* |  | -5.45E-04 | 2.62E-04 | 3.80E-02 | 4.57E-01 | 2.77E+00 | 8.69E-01 |
| cg05897122 | 4 | 52246540 | *NA* |  | -3.89E-05 | 2.53E-04 | 8.78E-01 | 4.39E+00 | 2.86E+00 | 1.26E-01 |
| cg22178392 | 5 | 151080128 | *TNIP1* |  | 2.52E-04 | 1.73E-04 | 1.46E-01 | 2.67E+00 | 4.10E+00 | 5.15E-01 |
| cg11001536 | 5 | 154110212 | *MFAP3* |  | -4.05E-04 | 3.61E-04 | 2.63E-01 | 3.07E-01 | 1.94E+00 | 8.75E-01 |
| cg24378227 | 6 | 1396535 | *NA* |  | 2.69E-04 | 1.97E-04 | 1.72E-01 | 3.43E+00 | 3.54E+00 | 3.33E-01 |
| cg03188976 | 7 | 134759112 | *CALD1* |  | 6.15E-05 | 2.72E-04 | 8.21E-01 | -2.64E+00 | 2.70E+00 | 3.29E-01 |
| cg00285394 | 8 | 124999711 | *SQLE* |  | 7.43E-04 | 3.83E-04 | 5.26E-02 | 3.63E+00 | 1.89E+00 | 5.44E-02 |
| cg14643264 | 9 | 27408243 | *MOB3B* |  | 4.34E-04 | 2.39E-04 | 6.99E-02 | 3.54E+00 | 3.00E+00 | 2.38E-01 |
| cg04999435 | 11 | 63463038 | *HRASLS5* |  | 8.27E-06 | 2.23E-04 | 9.70E-01 | -1.27E-01 | 3.18E+00 | 9.68E-01 |
| cg15705971 | 11 | 92377095 | *FAT3* |  | 1.13E-04 | 1.45E-04 | 4.35E-01 | 1.08E+00 | 4.91E+00 | 8.26E-01 |
| cg03308494 | 12 | 57619483 | *AC025165.8;SLC26A10* |  | -3.04E-04 | 1.99E-04 | 1.27E-01 | -7.04E-01 | 3.71E+00 | 8.49E-01 |
| cg08261450 | 12 | 129900188 | *TMEM132D* |  | 3.77E-05 | 1.51E-04 | 8.03E-01 | 1.08E+01 | 4.67E+00 | 2.07E-02 |
| cg20448706 | 14 | 32472634 | *AKAP6* |  | 1.09E-04 | 1.68E-04 | 5.16E-01 | 7.53E+00 | 4.45E+00 | 9.12E-02 |
| cg26874872 | 16 | 26145040 | *NA* |  | -6.99E-05 | 1.54E-04 | 6.50E-01 | 5.60E+00 | 4.76E+00 | 2.40E-01 |
| cg10508349 | 16 | 68908327 | *TANGO6* |  | -1.84E-04 | 3.10E-04 | 5.52E-01 | 9.02E-01 | 2.42E+00 | 7.10E-01 |
| cg10001987 | 17 | 7478449 | *ZBTB4* |  | 4.47E-04 | 4.18E-04 | 2.84E-01 | -1.45E+00 | 1.71E+00 | 3.97E-01 |

Abbreviations: chr_hg38, chromosome in GRCh38/hg38; Start_hg38, CpG start position in GRCh38/hg38.

*The CpGs was epigenome-wide significant in this study (*P* < 9x10^-8^).

Additional file 1: Table S8. The overlapping nearby genes of the significant lipid levels related CpGs between our findings and the ones previously reported in the general population in EWAS Catalog.

| SJLIFE CpGs | Overlapping Genes | SJLIFE EWAS |
| --- | --- | --- |
| cg00574958 | *CPT1A* | EA TC exposure; EA TG exposure |
| cg17058475 | *CPT1A* | EA TC exposure; EA TG exposure |
| cg05325763 | *CPT1A* | EA TC exposure; EA TG exposure |
| cg09737197 | *CPT1A* | EA TG exposure |
| cg16740586 | *ABCG1* | EA TG exposure |
| cg10886794 | *AXIN1* | EA TG exposure |
| cg19120513 | *BIRC3* | EA TG exposure |
| cg10417863 | *GOLGA3* | EA TG exposure |
| cg01620154 | *HDAC7* | EA TG exposure |
| cg09097291 | *PSMF1* | EA TG exposure |
| cg03725309 | *SARS* | EA TG exposure |
| cg07507418 | *TRERF1* | EA TG exposure |
| cg14543017 | *PCIF1* | EA HDL exposure |
| cg18966688 | *AUTS2* | AA TG exposure |
| cg05365685 | *ATP2B2* | EA TG outcome |
| cg13062424 | *C12orf65* | EA TG outcome |
| cg05453148 | *DAPK1* | EA TG outcome |
| cg01758046 | *ECE1* | EA TG outcome |
| cg20203534 | *GMDS* | EA TG outcome |
| cg09492247 | *GPC5* | EA TG outcome |
| cg24040155 | *LRP5* | EA TG outcome |
| cg20703271 | *MAPKBP1* | EA TG outcome |
| cg13522114 | *MLLT1* | EA TG outcome |
| cg19884600 | *PYHIN1* | EA TG outcome |
| cg10101263 | *SNX19* | EA TG outcome |
| cg20138617 | *TLL2* | EA TG outcome |

Additional file 1: Table S9. Previous EWAS results (reported in EWAS Catalog with *P*<9x10^-8^ and tissue including blood) for 15 additional blood lipids associated CpG hits identified in the meta-EWAS.

| CpG | Chr | Pos | Gene | Beta | SE | *P* | Trait | PMID | | Tissue |
| --- | --- | --- | --- | --- | --- | --- | --- | --- | --- | --- |
| cg23550429 | 2 | 31020802 | *CAPN13* | NA | NA | 1.67E-21 | Age 4 vs age 0 | 28056824 | Whole blood, cord blood | |
| cg23550429 | 2 | 31020802 | *CAPN13* | 0.03955 | 0.00456 | 1.70E-15 | Tissue | 30602389 | Buccal cells and peripheral blood mononuclear cells | |
| cg23550429 | 2 | 31020802 | *CAPN13* | -0.003 | 0.00016 | 0 | age | 33450751 | Whole blood | |
| cg23550429 | 2 | 31020802 | *CAPN13* | 0.0042 | 0.001 | 5.00E-05 | age | 33450751 | Whole blood | |
| cg27324117 | 4 | 154714287 | *-* | NA | NA | 3.19E-43 | Age 4 vs age 0 | 28056824 | Whole blood, cord blood | |
| cg27324117 | 4 | 154714287 | *-* | -0.39577 | 0.00666 | 5.10E-126 | Tissue | 30602389 | Buccal cells and peripheral blood mononuclear cells | |
| cg27324117 | 4 | 154714287 | *-* | 0.00047 | 0.00012 | 8.10E-05 | Age | 23177740 | Whole blood | |
| cg27324117 | 4 | 154714287 | *-* | 0.0037 | 0.00014 | 0 | age | 33450751 | Whole blood | |
| cg27324117 | 4 | 154714287 | *-* | -0.0045 | 0.00098 | 5.00E-06 | age | 33450751 | Whole blood | |
| cg27324117 | 4 | 154714287 | *-* | 2.047 | 0.499 | 6.60E-05 | ADHD | 33184255 | Cord Blood | |
| cg01082498 | 11 | 68608225 | *CPT1A* | -38.42 | 9.41 | 4.98E-05 | Postprandial lipemia | 27777315 | CD4+ T-cells | |
| cg01082498 | 11 | 68608225 | *CPT1A* | -61.31 | 12.61 | 1.83E-06 | Postprandial lipemia | 27777315 | CD4+ T-cells | |
| cg01082498 | 11 | 68608225 | *CPT1A* | -43.83 | 7.34 | 3.33E-09 | Postprandial lipemia | 27777315 | CD4+ T-cells | |
| cg01082498 | 11 | 68608225 | *CPT1A* | -0.008 | 0.00124 | 1.06E-10 | Very low-density lipoprotein cholesterol | 24920721 | CD4+ T-cells | |
| cg01082498 | 11 | 68608225 | *CPT1A* | -0.011 | 0.00163 | 8.79E-11 | Triglycerides | 24920721 | CD4+ T-cells | |
| cg01082498 | 11 | 68608225 | *CPT1A* | 0.0273 | 0.0021 | 4.10E-28 | Tissue | 30602389 | Buccal cells and peripheral blood mononuclear cells | |
| cg01082498 | 11 | 68608225 | *CPT1A* | NA | NA | 3.80E-05 | Alcohol consumption | 31789449 | Whole blood | |
| cg01082498 | 11 | 68608225 | *CPT1A* | NA | NA | 2.20E-07 | Alcohol consumption | 31789449 | Whole blood | |
| cg01082498 | 11 | 68608225 | *CPT1A* | 0.00052 | 7.40E-05 | 2.70E-12 | age | 33450751 | Whole blood | |
| cg01082498 | 11 | 68608225 | *CPT1A* | 0.00063 | NA | 3.80E-17 | age | 33450751 | Whole blood | |
| cg01082498 | 11 | 68608225 | *CPT1A* | -8.00E-04 | 0.00019 | 2.30E-05 | Obesity | 32788176 | Whole blood | |
| cg19590858 | 14 | 35009852 | *EAPP* | -0.0024 | 0.00016 | 0 | age | 33450751 | Whole blood | |
| cg19590858 | 14 | 35009852 | *EAPP* | 0.0015 | NA | 1.30E-17 | age | 33450751 | Whole blood | |
| cg11686214 | 19 | 59084984 | *MZF1;LOC100131691* | -0.00015 | 1.60E-05 | 0 | age | 33450751 | Whole blood | |
| cg11686214 | 19 | 59084984 | *MZF1;LOC100131691* | 0.0024 | 0.00041 | 3.10E-09 | Schizophrenia | 33279932 | Whole blood | |
| cg11686214 | 19 | 59084984 | *MZF1;LOC100131691* | NA | NA | 3.60E-09 | Schizophrenia | 33279932 | Whole blood | |

Abbreviations: ADHD, attention-deficit and hyperactivity disorder.

Additional file 1: Figure S1. Quantile-Quantile plots of exposure and outcome EWAS on lipids among survivors of European ancestry.

Abbreviations: EWAS, epigenome-wide association study; GIF, genomic inflation factors; HDL, high-density lipoprotein; LDL, low-density lipoprotein; TG, triglycerides; TC, total cholesterol.

Additional file 1: Figure S2. Quantile-Quantile plots of exposure and outcome EWAS on lipids among survivors of African ancestry.

Abbreviations: EWAS, epigenome-wide association study; GIF, genomic inflation factors; HDL, high-density lipoprotein; LDL, low-density lipoprotein; TG, triglycerides; TC, total cholesterol.

Additional file 1: Figure S3. Venn plots of significant CpGs identified through EWAS on lipids among survivors of European ancestry.

Abbreviations: EWAS, epigenome-wide association study; HDL, high-density lipoprotein; LDL, low-density lipoprotein; TG, triglycerides; TC, total cholesterol.

Additional file 1: Figure S4. Manhattan plots of blood lipids EWAS among survivors of African ancestry in SJLIFE.

Abbreviations: EWAS, epigenome-wide association study; HDL, high-density lipoprotein; LDL, low-density lipoprotein; TG, triglycerides; TC, total cholesterol.

Additional file 1: Figure S5. A flow chat of 2572 annotated genes related to blood lipids level in GWAS Catalog and EWAS Catalog.

Abbreviations: HDL, high-density lipoprotein; LDL, low-density lipoprotein; TG, triglycerides; TC, total cholesterol.
